# Light‐Activated Anti‐Vascular Combination Therapy against Choroidal Neovascularization

**DOI:** 10.1002/advs.202404218

**Published:** 2024-08-29

**Authors:** Shuting Xu, Jia Li, Kaiqi Long, Xiaoling Liang, Weiping Wang

**Affiliations:** ^1^ State Key Laboratory of Pharmaceutical Biotechnology The University of Hong Kong Hong Kong 999077 China; ^2^ Department of Pharmacology and Pharmacy Li Ka Shing Faculty of Medicine The University of Hong Kong Hong Kong 999077 China; ^3^ Laboratory of Molecular Engineering and Nanomedicine Dr. Li Dak‐Sum Research Centre The University of Hong Kong Hong Kong 999077 China; ^4^ State Key Laboratory of Ophthalmology Zhongshan Ophthalmic Center Sun Yat‐sen University Guangdong Provincial Key Laboratory of Ophthalmology and Visual Science Guangzhou 510060 China

**Keywords:** choroidal neovascularization (CNV), combretastatin A‐4, cyanine prodrug, near‐infrared (NIR) light‐activation, self‐assembled nanoparticles

## Abstract

Choroidal neovascularization (CNV) underlies the crux of many angiogenic eye disorders. Although medications that target vascular endothelial growth factor (VEGF) are approved for treating CNV, their effectiveness in destroying new blood vessels is limited, and invasive intravitreal administration is required. Additionally, other drugs that destroy established neovessels, such as combretastatin A‐4, may have systemic side effects that limit their therapeutic benefits. To overcome these shortcomings, a two‐pronged anti‐vascular approach is presented for CNV treatment using a photoactivatable nanoparticle system that can release a VEGF receptor inhibitor and a vascular disrupting agent when irradiated with 690 nm light. The nanoparticles can be injected intravenously to enable anti‐angiogenic and vascular disrupting combination therapy for CNV through light irradiation to the eyes. This approach can potentiate therapeutic effects while maintaining a favorable biosafety profile for choroidal vascular diseases.

## Graphical abstract

Near‐infrared light‐triggered prodrug activation strategy is utilized for anti‐angiogenic and vascular disrupting combination therapy of choroidal neovascularization (CNV). The intravenously administrated photoactivatable nanoparticles can achieve a significant reduction of CNV lesions, without any noticeable side effects in vivo. This approach holds the potential for the accurate delivery of dual‐function anti‐vascular agents to neovascular lesions.

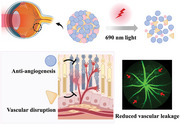

## 1 Introduction

Choroidal neovascularization (CNV) is featured by the aberrant growth of choroidal vessels into retinal pigment epithelium (RPE) or subretinal space.^[^ ^1^, ^2^ ^]^ It represents the major hallmark of many sight‐threatening angiogenic disorders, particularly exudative age‐related macular degeneration (AMD).^[^ ^3^ ^]^ Notably, exudative AMD accounts for over 90% of cases where AMD patients experience irreversible visual impairment.^[^ ^4^ ^]^ It is anticipated that ≈288 million people will be affected by AMD by 2040, which may increase along with the population aging.^[^ ^5^ ^]^ Currently, the primary treatment for CNV is anti‐vascular endothelial growth factor (VEGF) therapy.^[^ ^6^ ^]^ However, its invasive intravitreal administration method often discourages patient compliance and even causes severe complications like traumatic cataracts and endophthalmitis.^[^ ^3^, ^7^, ^8^ ^]^ Other unresolved issues of anti‐VEGF therapy include its limited efficacy in regressing existing neovessels, which can damage retinal layers, potentially leading to recurrent bleeding and permanent vision loss.^[^ ^9^, ^10^, ^11^ ^]^ One promising treatment option for CNV is the combination of anti‐VEGF medications with vascular disrupting agents that occlude the vessels. Combretastatin A‐4 (CA4) is a representative vascular disrupting drug candidate for vessel ablation. As a tubulin‐binding agent, CA4 inhibits the polymerization of microtubules of endothelial cells, which further leads to cytoskeletal disruption and CNV regression.^[^ ^12^, ^13^ ^]^ However, systemic administration of CA4 poses the risks of cardiovascular events.^[^ ^14^ ^]^ There is a need to develop novel drug delivery systems that can deliver anti‐vascular agents precisely to the posterior side of the eyes while minimizing both localized and systemic side effects.

Stimuli‐responsive nanosystems have shown immense potential in delivering drugs to the posterior eye segment by enabling tunable drug release in target lesions.^[^ ^15^, ^16^, ^17^, ^18^ ^]^ Due to the noninvasive nature and high spatiotemporal controllability of light irradiation, as well as its ability to penetrate through the eyeball, using light irradiation to control drug release in the eyes is an appealing approach.^[^ ^19^ ^]^ Many photocleavable protection groups (PPGs), such as coumarin and boron‐dipyrromethene, have been utilized to mask the bioactive moieties of drug molecules or targeting ligands.^[^ ^20^, ^21^, ^22^ ^]^ Upon light irradiation at specific wavelengths, these PPGs can be removed, allowing fine control over drug release profiles or targeting properties.^[^ ^19^, ^23^, ^24^ ^]^ Of these, cyanine‐caged nanoplatforms with near‐infrared (NIR) light responsiveness (650–900 nm) have been widely exploited to achieve controllable drug release.^[^ ^25^, ^26^, ^27^ ^]^ They were well‐suited for ophthalmologic applications, given the improved tissue penetration and biocompatibility of NIR light compared to short‐wavelength light (e.g., UV or blue light).^[^ ^28^ ^]^ The NIR light‐triggered uncaging approach could enable a comparatively safe and convenient administration method for CNV treatment. This method involves intravenous injection of photocaged agents and triggering drug release at the back of the eyes.

**Figure 1:**
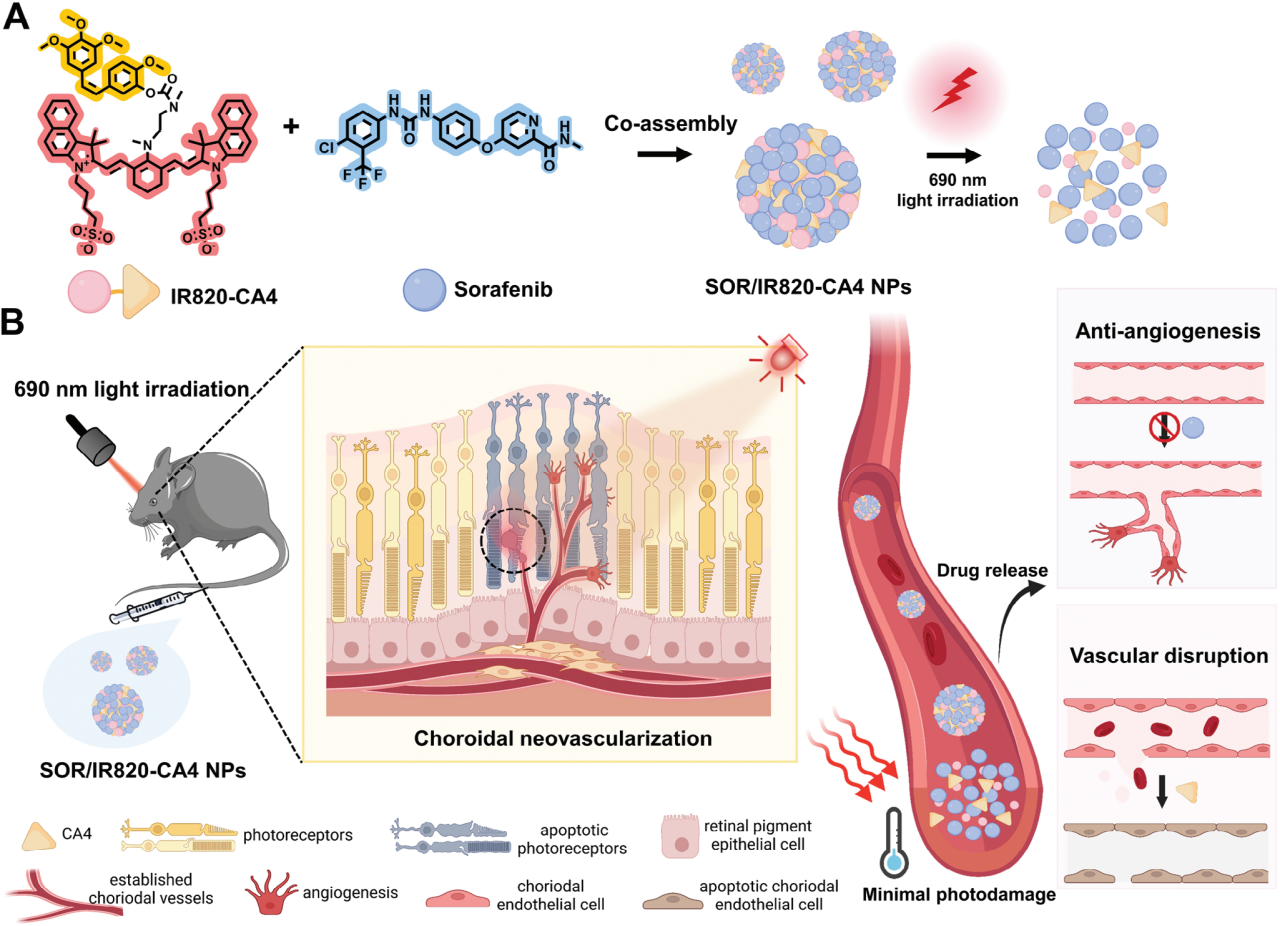
A) Schematic representation of the co‐assembly of SOR/IR820‐CA4 NPs and near‐infrared (NIR)‐light‐triggered nanoparticle disassembly. B) Schematic illustration showing the mechanism of SOR/IR820‐CA4 NPs for the anti‐vascular combination treatment of choroidal neovascularization (CNV). After intravenous injection of SOR/IR820‐CA4 NPs in a laser‐induced CNV mouse model, 690 nm light irradiation to the mouse eye triggers the release of the anti‐vascular drugs, with sorafenib (SOR) inhibiting angiogenesis and combretastatin A‐4 (CA4) devastating established CNV vasculature.

In this work, we developed a two‐pronged anti‐vascular strategy for intravenous treatment of CNV using photoactivatable nanoparticles (**Figure** **1**). Our approach involves the synthesis of a NIR light‐cleavable cyanine prodrug (IR820‐CA4) of vascular disrupting agent CA4. The co‐assembly of this prodrug with the VEGF receptor inhibitor sorafenib (SOR) forms a nanosystem, hereafter denoted as SOR/IR820‐CA4 NP. We demonstrated that NIR‐light irradiation (690 nm) triggered the photolysis of IR820‐CA4 prodrug and nanoparticle disassembly, leading to the release of anti‐angiogenic drug SOR and vascular disrupting agent CA4. Therefore, after intravenous injection of SOR/IR820‐CA4 NPs into a laser‐induced CNV mouse model and applying light irradiation to the mouse eyes, NIR light‐triggered release of the two drugs confers a robust CNV suppression. Importantly, the treatment did not cause any noticeable systemic side effects or localized photodamage effects. Collectively, these results demonstrated the potential of this NIR‐light‐activatable nanosystem for delivering synergistic anti‐vascular drugs to CNV lesions with enhanced therapeutic efficacy.

## 2 Results

### 2.1 Synthesis and Characterization of IR820‐CA4 Prodrug

**Figure 2:**
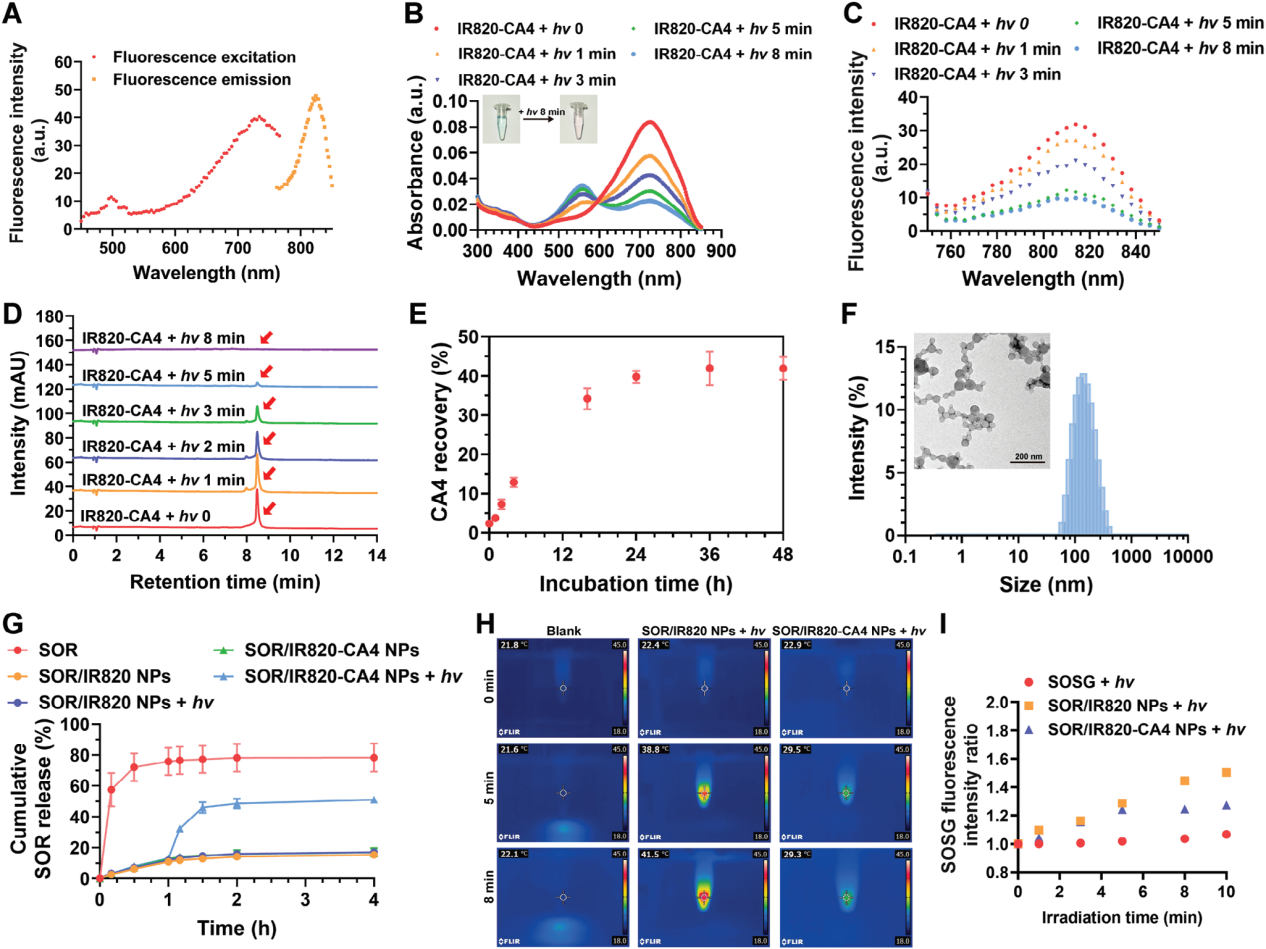
Characterization of IR820‐CA4 prodrug and SOR/IR820‐CA4 NPs. A) Representative fluorescence emission and excitation spectra of IR820‐CA4. B) Representative absorption spectra and photographs of IR820‐CA4 solution upon exposure to near‐infrared (NIR) light irradiation for varying periods (690 nm, 80 mW cm^−2^). C) Representative fluorescence spectra of IR820‐CA4 upon exposure to 690 nm light irradiation for varying periods (80 mW cm^−2^). Ex = 735 nm. D) Representative HPLC chromatograms of IR820‐CA4 after receiving different periods of 690 nm light irradiation (80 mW cm^−2^). Red arrows denote the gradual decline of IR820‐CA4 content upon light irradiation. E) Quantitative analysis of CA4 recovery percentages at different time points. IR820‐CA4 solution was exposed to 690 nm light irradiation (80 mW cm^−2^, 8 min) and then incubated at 37 °C, and stirred at 120 rpm for various periods. F) Representative size distribution histogram and transmission electron microscopy (TEM) image of SOR/IR820‐CA4 NPs. G) Cumulative SOR release profiles of SOR/IR820 NPs and SOR/IR820‐CA4 NPs. The nanoparticle solutions were exposed to 690 nm light irradiation (80 mW cm^−2^, 5 min) at 1 h or not. H) Representative infrared thermal images of SOR/IR820 NP and SOR/IR820‐CA4 NP solutions exposed to 690 nm laser (80 mW cm^−2^) for various periods. I) Variations of SOSG fluorescence intensity ratios of SOR/IR820 NPs and SOR/IR820‐CA4 NPs after different durations of 690 nm light irradiation (80 mW cm^−2^). SOSG fluorescence intensity ratios were calculated relative to the initial time point. Data were presented as mean ± standard deviation. *n = *3.

The synthesis of a novel photocaged prodrug IR820‐CA4 was accomplished in three steps based on the previous literature,^[^ ^29^ ^]^ and the procedures were described in Figure S1 (Supporting Information). The purified product was ascertained by proton nuclear magnetic resonance (^1^H NMR) spectroscopy and mass spectroscopy (MS) (Figure S2, Supporting Information). Molecularly dissolved IR820‐CA4 exhibited a broadened and intense absorption band ≈710 nm (Figure S3, Supporting Information). The maximum excitation wavelength (Ex) and fluorescence emission wavelength (Em) of IR820‐CA4 were found near 735 and 825 nm, respectively (**Figure** **2**A).

According to the previous reports, cyanine‐caged prodrugs undergo photooxidative cleavage and cyclization steps to release the masked drug molecules under light irradiation.^[^ ^28^, ^30^ ^]^ Therefore, we investigated the changes in the photophysical property of IR820‐CA4 upon NIR light irradiation at 690 nm (80 mW cm^−2^). As shown in Figure 2B,C, the NIR absorption peak of IR820‐CA4 gradually decreased along with the prolonged irradiation periods, accompanied by a decline in its fluorescence intensity. The blue color of the IR820‐CA4 solution also transformed to purple after light irradiation. We assumed that the dramatic blueshift and fluorescence quenching of IR820‐CA4 was due to the photolysis of the prodrug. Afterward, we confirmed the light‐triggered degradation of IR820‐CA4 using high‐performance liquid chromatography (HPLC). As shown in Figures 2D and S4 (Supporting Information), the prodrug concentration steadily decreased upon exposure to light irradiation (690 nm, 80 mW cm^−2^), and nearly complete degradation of the prodrug was observed at 5 min post‐irradiation. In contrast, > 80% of IR820 dye remained upon light irradiation (690 nm, 80 mW cm^−2^, 8 min) (Figure S5, Supporting Information). After that, the prodrug solution was incubated at 37 °C for varying periods, where the photolyzed products and rearranged intermediates could be detected by liquid chromatography‐mass spectroscopy (LC/MS) (Figures S6 and S7, Supporting Information). We found that the recovery yield of CA4 could reach ≈42% at 36 h post‐irradiation (Figure 2E). These results reveal that CA4 can be efficiently released from the designed prodrug under NIR light irradiation at 690 nm.

### 2.2 Preparation and Characterization of SOR/IR820‐CA4 NPs

We fabricated the prodrug‐based nanoparticles with the nanoprecipitation method. Of note, IR820‐CA4 and SOR could self‐assemble into nanoparticles without extra excipients, consistent with previous reports that cyanine dyes could serve as stabilizers for nano‐formulations.^[^ ^22^, ^31^ ^]^ Molecular docking results implied that intermolecular interactions exist between SOR and IR820‐CA4, including π–π stacking and hydrogen bonding, which might contribute to the self‐assembly (Figure S8, Supporting Information). The optimized formulation, thereafter marked as SOR/IR820‐CA4 NPs, was attained at the 5% (w/w) of IR820‐CA4 relative to the total feeding amount of SOR according to the size distribution and the prodrug loading capacity (Figure S9, Table S1, Supporting Information). The spherical shape of SOR/IR820‐CA4 NPs was observed by transmission electron microscopy (TEM) imaging (Figure 2F), with a hydrodynamic particle size of ≈120 nm and a polydispersity index (PDI) of 0.12. SOR/IR820‐CA4 NPs showed an obvious redshift of the characteristic absorption peak to ≈750 nm (Figure S10, Supporting Information). The drug loading capacity of SOR/IR820‐CA4 NPs was 2.9% and 97.1% for the prodrug and SOR, respectively. After dispersion in 10% fetal bovine serum (FBS)‐containing cell culture media at 37 °C for 48 h, there was no dramatic size change for SOR/IR820‐CA4 NPs, suggesting their good colloidal ability in vitro (Figure S11, Supporting Information). We also fabricated the nanoparticles (SOR/IR820 NPs) comprising cyanine photocage IR820 instead of IR820‐CA4 prodrug, which displayed similar physicochemical properties to SOR/IR820‐CA4 NPs (Table S1).

### 2.3 NIR Light‐triggered Release of SOR and Phototoxicity of SOR/IR820‐CA4 NPs

To assess the NIR‐light responsiveness of the prodrug‐based nanoparticles, we first examined the size changes of both nanoparticles upon light irradiation (690 nm, 80 mW cm^−2^, 10 min). In contrast to negligible size variations observed in SOR/IR820 NP solution, there was a dramatic size increase for SOR/IR820‐CA4 NPs following light exposure, indicating the disassembly of nanoparticles and formation of aggregates (Figure S12, Supporting Information). Subsequently, we investigated NIR light‐triggered SOR release using the dialysis method. As revealed in Figures 2G and S13 (Supporting Information), both nanoparticles exhibited a slow release of SOR in the dark initially, whereas applying the light irradiation (690 nm, 80 mW cm^−2^, 5 min) at 1 h caused a burst SOR release from SOR/IR820‐CA4 NPs, but not SOR/IR820 NPs. The cumulative SOR release of irradiated SOR/IR820‐CA4 NPs was ≈51% at 4 h, ≈2.98 times higher than SOR/IR820 NPs plus light irradiation or SOR/IR820‐CA4 NPs in the dark (≈17.1%). Such NIR light‐triggered drug release profiles of SOR/IR820‐CA4 NPs can be explained by the photolabile property of the cyanine prodrug, indicating that NIR light irradiation can lead to nanoparticle disassembly and accelerated SOR release.

Many cyanine derivatives can serve as photosensitive agents for inducing photothermal therapy (PTT) or photodynamic therapy (PDT).^[^ ^32^ ^]^ However, these traits are undesirable for this NIR light‐activatable nanosystem, which exerts therapeutic functions without relying on phototoxicity. Therefore, we studied the photo‐damaging effect of SOR/IR820‐CA4 NPs in vitro. Noteworthily, we found the limited PTT effect of SOR/IR820‐CA4 NPs upon light irradiation (Figure 2H; Figure S14 Supporting Information), owing to the photodegradation of IR820 in SOR/IR820‐CA4 NPs (Figures S6 and S7, Supporting Information). As a comparison, cyanine dye‐incorporated nanoparticles, SOR/IR820 NPs, exhibited a time‐dependent increase in solution temperature (690 nm, 80 mW cm^−2^) upon light irradiation. The compromised PTT effect of the prodrug nanoparticles could prevent hyperthermia and retinal burns during accidental extended light exposure. Besides, photooxidation is reported as the primary photolysis pathway of diethylamine‐tethered heptamethine cyanines, where singlet oxygen (^1^O_2_) is required for the cleavage of cyanine polyene.^[^ ^33^ ^]^ Accordingly, we assumed that the photolysis of the prodrug by the produced ^1^O_2_ might lower reactive oxygen species (ROS) level from the prodrug nanoparticles. As proof of concept, we detected ^1^O_2_ generation from SOR/IR820‐CA4 NP solution upon light irradiation using a singlet oxygen sensor green (SOSG) probe (690 nm, 80 mW cm^−2^). SOR/IR820‐CA4 NPs showed an attenuated increase in SOSG fluorescence intensity after 5 min of light irradiation, likely attributed to the photo‐instability of cyanine prodrug and ^1^O_2_ consumption during the photolysis process (Figure 2I). In contrast, the SOSG fluorescence of the SOR/IR820 NP solution became intensified along with the prolonged irradiation time. These results together demonstrate the apparently reduced phototoxicity of SOR/IR820‐CA4 NPs.

### 2.4 Photoactivatable Anti‐Angiogenesis and Cytoskeleton Destruction

Before in vitro studies, we investigated the cellular uptake efficiency of SOR/IR820‐CA4 NPs with human umbilical vein endothelial cells (HUVECs), which is one of the common endothelial cell lines for anti‐angiogenesis research.^[^ ^34^ ^]^ HUVECs incubated with the prodrug nanoparticles exhibited a stronger fluorescence, ≈3.6 times higher than those treated with free IR820‐CA4 at 4 h post‐incubation (Figure S15, Supporting Information). The results were in accordance with the previous literature that nanoparticle formulations improved the cellular internalization of cyanine dyes.^[^ ^35^ ^]^

**Figure 3:**
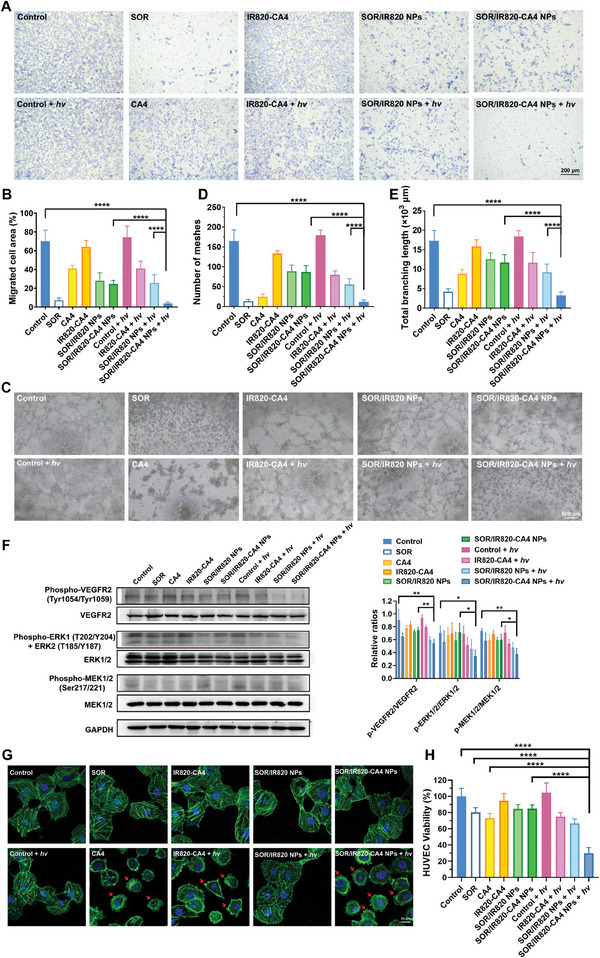
In vitro therapeutic evaluation of SOR/IR820‐CA4 NPs. A) Representative images of transwell migration assay of SOR/IR820‐CA4 NPs. Human umbilical vein endothelial cell (HUVEC) suspensions were incubated with 20 ng mL^−1^ VEGF_165_ and different formulations and then exposed to 690 nm light irradiation at 4 h (80 mW cm^−2^, 5 min). Cell migration to the bottom side of the transwell was observed at 12 h post‐irradiation. B) Quantitative analysis of migrated cell area per field of transwell migration assay. *n = *6. C) Representative images of tube formation assay of SOR/IR820‐CA4 NPs. HUVECs were treated with 20 ng mL^−1^ VEGF_165_ and various formulations, followed by 690 nm light irradiation or not (80 mW cm^−2^, 5 min). Images of tube formation were taken at 4 h post‐incubation. D) Quantitative analysis of the number of meshes and E) total branching length of HUVEC tubes of tube formation assay. *n = *5. F) Representative images and the quantification results of Western‐blot analysis of pro‐angiogenic signaling in HUVECs after SOR/IR820‐CA4 NP treatment. HUVECs were treated with 20 ng mL^−1^ VEGF_165_ and different formulations for 24 h, with or without 690 nm light irradiation (80 mW cm^−2^, 5 min). *n = *3. G) Representative confocal images of Alexa Fluor 488‐phalloidin‐stained cytoskeleton of HUVECs after SOR/IR820‐CA4 NP treatment. HUVECs were incubated with different formulations followed by 690 nm light irradiation (80 mW cm^−2^, 5 min) or not. Red arrows denote severe cytoplasm contraction and membrane blebbing. H) Quantitative HUVEC viability after various treatments. *n = *6. Data were presented as mean ± standard deviation. * *p* < 0.05; ** *p* < 0.01; **** *p* < 0.0001.

To explore the light‐potentiated anti‐angiogenic effect of SOR/IR820‐CA4 NPs, we first assayed for essential angiogenic activities of HUVECs, including cell migration and tube formation. As shown in **Figure** **3**A,B, the migration area of HUVECs was decreased by free CA4 (40.9% of migrated cell area per field) and SOR (7.2% of migrated cell area per field) to different extents. Conversely, IR820‐CA4 prodrug without light irradiation showed no evidence of inhibiting VEGF‐induced cell migration (91.3% relative to the control), indicating undermined anti‐angiogenic ability of the prodrug. Notably, SOR/IR820‐CA4 NPs plus light irradiation nearly blocked the HUVEC migration (3.8% of migrated cell area per field). Likewise, the inhibitory effect of SOR/IR820‐CA4 NPs plus light irradiation on tube differentiation was the strongest of all groups, causing a substantial decline in the number of meshed and total branching length to 6.8% and 18.9%, respectively, relative to the control (Figure 3C–E). Whereas SOR/IR820‐CA4 NPs without light irradiation cannot block HUVEC tube formation, where the number of meshes and total branching length were reduced to 52.7% and 67.7%, respectively, relative to the control. Such prominent anti‐angiogenic effect of SOR/IR820‐CA4 NPs plus light irradiation could be credited to NIR light‐triggered nanoparticle disassembly to release the two‐pronged anti‐vascular drugs.

Furthermore, to elucidate the photoactivatable anti‐angiogenic mechanism of SOR/IR820‐CA4 NPs, we detected angiogenic signaling of HUVECs exposed to different formulation treatments. We observed that free SOR attenuated the phosphorylation of vascular endothelial growth factor receptor 2 (VEGFR2), extracellular signal‐regulated kinases 1/2 (ERK1/2), and MEK 1/2 to 72.2%, 79.8%, and 79.3%, respectively, relative to the control (Figure 3F). These results were in line with the reports that SOR inhibits angiogenic cascades by blocking VEGFR/MEK/ERK pathways, which are pivotal in multiple angiogenic processes, including endothelial cell proliferation and migration.^[^ ^36^, ^37^ ^]^ Notably, SOR/IR820‐CA4 NPs plus light irradiation treatment (690 nm, 80 mW cm^−2^, 5 min) considerably downregulated the phosphorylation levels of these angiogenic markers to 60.7%, 48.7%, and 51.3%, separately, relative to the control (Figure 3F). In comparison, SOR/IR820‐CA4 NPs without light irradiation were apparently less effective in blocking VEGFR/MEK/ERK cascades (83.3%, 94.1%, and 82.0%, respectively, relative to the control). The results indicate that NIR‐light‐triggered release of SOR might contribute to the potentiated anti‐angiogenic effect of SOR/IR820‐CA4 NPs.

CA4 is a well‐known tubulin‐binding agent that can inhibit the polymerization of microtubules of endothelial cells, ultimately leading to cytoskeletal disruption and CNV regression.^[^ ^12^, ^38^ ^]^ Therefore, we investigated the effect of SOR/IR820‐CA4 NPs on cytoskeletal integrity by Alexa Fluor 488‐conjugated phalloidin staining of actin. As revealed in Figure 3G, HUVECs exposed to the treatments of SOR, IR820‐CA4, and SOR/IR820‐CA4 NPs without light irradiation displayed orderly microfilament assembly and intact cytoskeleton, which is similar to the control. These phenomena indicate that SOR could not damage the cytoskeleton, nor did IR820‐CA4 prodrug. In comparison, light irradiation (690 nm, 80 mW cm^−2^, 5 min) to SOR/IR820‐CA4 NP‐treated cells resulted in severe cytoplasmic contraction and membrane blebbing. It also occurred when HUVECs were treated with the equivalent concentration of 10 nm CA4. Overall, it was evident that the vascular disrupting capacity of SOR/IR820‐CA4 NPs can be recovered by NIR light irradiation at 690 nm.

### 2.5 In Vitro Cytotoxicity and Biocompatibility Evaluation of SOR/IR820‐CA4 NPs

Given the different actions of the anti‐angiogenic drug SOR and vascular disrupting agent CA4, we next sought to evaluate whether this drug combination conferred synergistic cytotoxicity effects on endothelial cells. Figure S16 (Supporting Information) showed a potential synergy between SOR and CA4 for killing endothelial cells, with an average synergistic index of 16.17 ± 2.72 within the equivalent concentration range of 1–20 µm SOR. Importantly, compared to IR820‐CA4 plus light irradiation (75.0% of cell viability) or SOR/IR820 NPs plus light irradiation treatments (66.7% of cell viability), SOR/IR820‐CA4 NPs plus light irradiation demonstrated superior cytotoxicity (29.7% of cell viability) (Figure 3H). These results raise the possibility that improved cellular uptake and the release of anti‐vascular drugs effectively enhance the cell‐killing effects of SOR/IR820‐CA4 NPs plus light irradiation. In addition, SOR/IR820‐CA4 NPs possessed desirable biocompatibility to human retinal pigment epithelial cells ARPE‐19 and HUVECs in the dark, as evidenced by high cell viability (≈80%) after 48 h of incubation with 40 µm SOR/IR820‐CA4 NPs (Figure S17, Supporting Information).

### 2.6 In Vivo Pharmacokinetics and Intraocular Distribution Profiles of SOR/IR820‐CA4 NPs

Before in vivo therapeutic assessments, we examined the pharmacokinetic properties of SOR/IR820‐CA4 NPs in mice. Figure S18 and Table S2 (Supporting Information) show that free IR820‐CA4 prodrug exhibited rapid plasma clearance after tail vein injection, with an estimated plasma half‐life of ≈2.80 h. In comparison, SOR/IR820‐CA4 NPs displayed an extended circulating duration (≈7.86 h), ≈1.58 and 2.81 times higher than the original plasma half‐lives of SOR and IR820‐CA4 prodrug, respectively. These results are also consistent with the previous literature that nanoparticle formulations can improve the pharmacokinetic properties of cyanine derivatives.^[^ ^39^ ^]^ Besides, we detected the intraocular drug concentrations at 1 h post‐intravenous administration of SOR solution or SOR/IR820‐CA4 NPs. As shown in Table S3, higher SOR concentrations were found in the choroid, RPE, and retina tissue (678.89–719.52 ng g^−1^) than in other ocular tissue, including vitreous body and cornea (≈162.69 ng g^−1^), in SOR/IR820‐CA4 NP group. The abundant choroidal bloodstream may facilitate nanoparticle perfusion into the CNV lesions and subsequent prodrug activation by light irradiation.^[^ ^40^, ^41^ ^]^

### 2.7 In vivo CNV Suppression by SOR/IR820‐CA4 NPs

Based on the in vitro data, we postulated that the combination of anti‐angiogenic agent SOR and microtubule‐depolymerizing agent CA4 might endow SOR/IR820‐CA4 NPs with light‐triggered dual ability to suppress neovascularization and devastate the CNV vessels. To verify this hypothesis, we investigated the in vivo therapeutic effects of SOR/IR820‐CA4 NPs on a laser‐induced CNV mouse model. After the induction of CNV by four laser injuries in each eye, the mice were subject to different treatments on days 3 and 5 and then studied by optical coherence tomography (OCT) on day 7. As a proof of concept, the cross–sectional OCT views showed a significant decrease in the central CNV thickness to 36.125 µm in the eyes treated with SOR/IR820‐CA4 NPs plus light irradiation (Figure S19, Supporting Information). However, CNV lesion thickness was prominent in other groups, including saline (94.5 µm), SOR (67.3 µm), SOR/IR820‐CA4 NPs (86.0 µm), and IR820‐CA4 plus light irradiation (59.8 µm). These results suggest that SOR/IR820‐CA4 NPs plus light irradiation attenuated the CNV activity more effectively compared to the sole anti‐vascular regimens.

**Figure 4:**
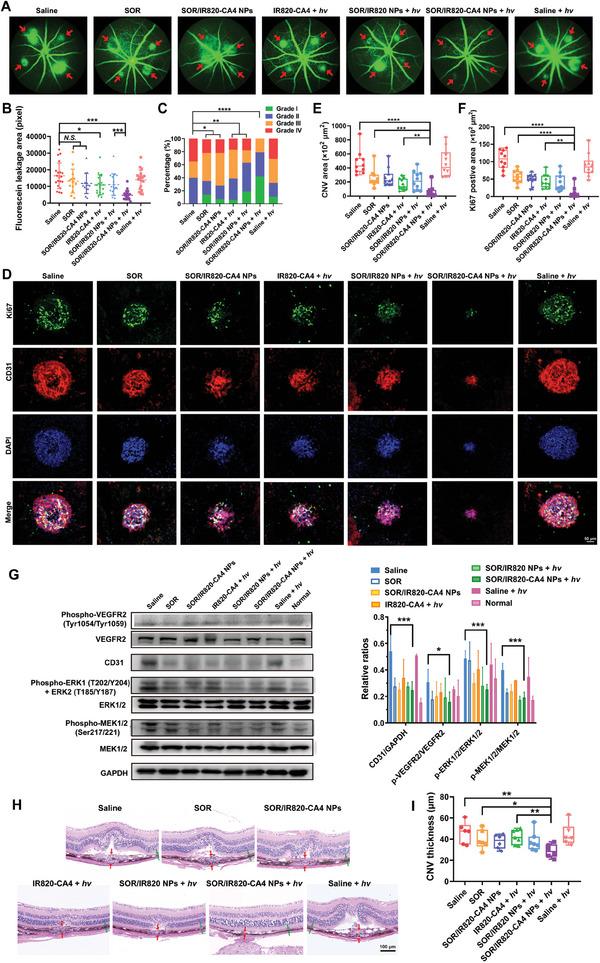
CNV suppression by SOR/IR820‐CA4 NPs in a laser‐induced CNV mouse model. A) Representative fundus fluorescein angiography (FFA) images of CNV mice on day 7. C57BL/6 mice were subjected to laser injury of the Bruch's membrane, and then intravenously injected with different formulations at the equivalent dose of 10 mg kg^−1^ SOR on day 3 and day 5, with or without 690 nm light irradiation (80 mW cm^−2^, 5 min) to the mouse eyes. B) Quantitative analysis of the fluorescence intensity of FFA results. *n = *14–20 lesions, 4–6 mice. C) Quantitative analysis of clinical grading results of fluorescein leakage. Grade I: No hyperfluorescence; Grade II: Hyperfluorescence without leakage; Grade III: Hyperfluorescence and late mild leakage; Grade IV: Bright hyperfluorescence with severe fluorescein leakage. *n = *14–20 lesions, 4–6 mice. D) Representative confocal images of co‐immunofluorescence staining of Ki67 and CD31 of RPE‐choroid flat‐mounts. Green, Ki67‐stained proliferating cell nuclei; Red, CD31‐stained vascular endothelial cells; Blue, DAPI‐stained nuclei. E) Quantitative analysis of CNV areas and F) Ki67‐positive proliferating cell areas. *n = *10–11 lesions, 4 mice. G) Representative images and the quantification results of Western‐blot analysis of pro‐angiogenic signaling of RPE‐choroid tissue from the CNV mice after different treatments. *n = *3. H) Representative hematoxylin and eosin (H&E)‐stained eye cross–sections from the CNV mice after different treatments. Red arrows denote CNV lesions and green arrows denote normal choroid. I) Quantitative analysis of the maximum CNV thickness. *n = *6–7 lesions. Data were presented as mean ± standard deviation. *N.S*., no statistical significance; * *p* < 0.05; ** *p* < 0.01; *** *p* < 0.001; **** *p* < 0.0001.

In addition, we examined the pathological vascular leakage of CNV mice by fundus fluorescein angiography (FFA). Generally, FFA images of the saline group exhibited extensive CNV leakage on day 7, with the mean fluorescein leakage area up to 16 394 pixels (**Figure** **4**A,B). The fluorescein leakage was still severe after the mice were treated with free SOR or SOR/IR820‐CA4 NPs without light irradiation. The mean fluorescein leakage area of these groups remained 80.4% and 72.0% relative to the control (13 181 and 11 800 pixels), respectively. Notably, SOR/IR820‐CA4 NPs plus light irradiation resulted in the most significant reduction in vessel leakiness, presenting the smallest fluorescein area of 28.8% relative to the control (4716 pixels). Meanwhile, the clinical grading results also confirmed that the predominant proportion (79%) of the lesion leakage grade of SOR/IR820‐CA4 NPs plus light irradiation group were altered to grade I‐II (no hyperfluorescence and hyperfluorescence without leakage) (Figure 4C). Despite that the distribution of lesion leakage grade was improved in other groups, there were still up to 21%, 17%, and 19% of CNV lesions at grade IV (bright hyperfluorescence with severe fluorescein leakage) after treatments with SOR, IR820‐CA4 plus light irradiation, and SOR/IR820 NPs plus light irradiation, respectively. To conclude, these results verify the robust protecting effect of SOR/IR820‐CA4 NPs plus light irradiation for reducing vascular leakage.

To quantify the CNV area and cell proliferation activity after the treatment, we next performed co‐immunofluorescence staining of CD31 and Ki67 on choroid complex flat‐mounts, which are representative markers for vascular endothelial cells and proliferating cells, respectively.^[^ ^42^ ^]^ As revealed in Figure 4D–F, the SOR/IR820‐CA4 NPs plus light irradiation group achieved the most efficient regression of CNV area after laser burn (61.39 × 10^2^ µm^2^), decreased by ≈87% compared to the saline group. By comparison, SOR or IR820‐CA4 plus light irradiation treatments only engendered moderate CNV suppression effect, reducing CNV dimensions by 46.3% and 65.6%, respectively. Meanwhile, SOR/IR820‐CA4 NPs plus light irradiation group also presented the fewest Ki67‐positive proliferating cell area (9.25 × 10^2^ µm^2^) compared to the groups of SOR (56.57 × 10^2^ µm), SOR/IR820‐CA4 NPs (48.67 × 10^2^ µm), and IR820‐CA4 prodrug plus light irradiation (43.55 × 10^2^ µm). Apart from these, SOR or IR820‐CA4 plus light irradiation were less effective at downregulating phosphorylation of ERK1/2 and MEK1/2 in the RPE‐choroid tissue, which remained up to 57.5% and 80.4% for the SOR group, and 86.8% and 83.3% for IR820‐CA4 plus light irradiation group relative to the control on day 7 (Figure 4G). While the phosphorylation of VEGFR2, ERK1/2, and MEK1/2 was greatly decreased to 51.8%, 47.9%, and 52.0%, respectively, in SOR/IR820‐CA4 NPs plus light irradiation group, along with a strong reduction in CD31 expression to 45.8% relative to the control. Histological examinations of eye cross–sections also showed that SOR/IR820‐CA4 NPs without light irradiation slightly reduce the maximum thickness of CNV lesions (37.7 µm) to ≈82% relative to the control (46.0 µm) (Figure 4H,I). Conversely, the SOR/IR820‐CA4 NPs plus light irradiation group exhibited an apparently smaller maximum CNV thickness of 28.9 µm, ≈63% relative to the control, which was in line with the cross–sectional OCT data. Overall, these findings clearly manifest that combining anti‐vascular approaches through angiogenesis inhibition and neovessel ablation offers significant advantages.

### 2.8 Biosafety of SOR/IR820‐CA4 NPs

**Figure 5:**
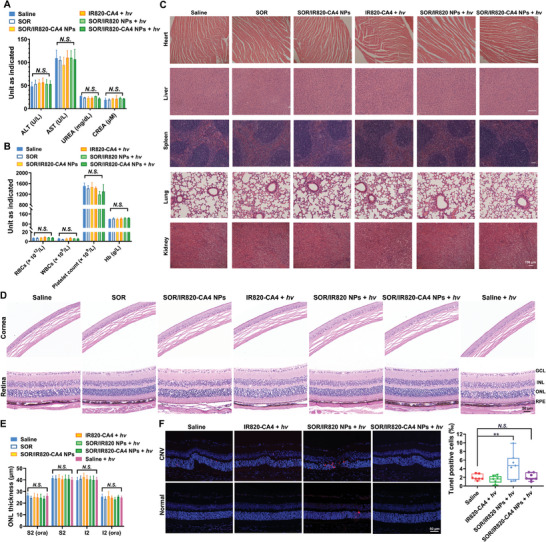
In vivo biosafety profiles of SOR/IR820‐CA4 NPs. CNV mice were administrated with different formulations at the equivalent dose of 10 mg kg^−1^ SOR on day 3 and day 5, with or without 690 nm light irradiation (80 mW cm^−2^, 5 min) to the mouse eyes. The mice were sacrificed on day 7 for biosafety evaluations. A) Hematological parameters and B) the results of routine blood count in CNV mice treated with different formulations. *n = *3. C) Representative H&E‐stained main organ sections of CNV mice after different formulation treatments. D) Representative H&E‐stained corneal and retinal sections of CNV mice after different formulation treatments. E) Quantitative analysis of the outer nuclear layer (ONL) thickness of H&E‐stained retinal sections of CNV mice after different formulation treatments. *n = *5. F) Representative confocal images and the quantification results of TUNEL‐stained retinal sections of CNV mice after different formulation treatments. Red, TUNEL‐stained apoptotic cell nuclei; Blue, DAPI‐stained nuclei. *n = *6–7. ALT, alanine aminotransferase; AST, aspartate aminotransferase; CREA, creatinine; RPE, retinal pigment epithelium; ONL, outer nuclear layer; INL, inner nuclear layer; GCL, ganglion cell layer; S2, superior quadrant; I2. inferior quadrant; ora, vicinity of ora serrata. Data were presented as mean ± standard deviation. *N.S*., no statistical significance; ** *p* < 0.01.

Favorable blood biocompatibility is one of the essential prerequisites for intravenously administrated nanoparticles.^[^ ^43^ ^]^ Therefore, after in vivo therapeutic evaluation, we evaluated the hemocompatibility profiles of SOR/IR820‐CA4 NPs. According to the data of serum biochemical assays in **Figure** **5**A, SOR/IR820‐CA4 NPs did not cause significant elevations in serum levels of alanine transaminase (ALT), aspartate transaminase (AST), urea and creatine (CREA), demonstrating that the prodrug nanoparticles posed no risks of impairing liver and kidney function. Besides, the blood parameters of SOR/IR820‐CA4 NP‐treated mice did not differ from those of saline‐treated mice, confirming good blood compatibility of the nanoparticles (Figure 5B). Moreover, there was no noticeable abnormality in the major organs of SOR/IR820‐CA4 NP‐treated mice, which further corroborates the biosafety of the nanoparticles (Figure 5C).

Additionally, we assessed the ocular biosafety of our proposed anti‐vascular strategy by histological analyses. Hematoxylin and eosin (H&E) photographs in Figure 5D revealed that the thickness of the cornea and retina was not significantly changed with or without the treatment with SOR/IR820‐CA4 NPs plus light irradiation. The quantitative analysis of the outer nuclear layer (ONL) thickness of SOR/IR820‐CA4 NP‐treated mice was also comparable to the control (Figure 5E). Moreover, we did not find evidence of retinal toxicity of SOR/IR820‐CA4 NPs plus light irradiation group, as proven by negligible TUNEL‐positive nuclei in the retina of the mouse eyes (Figure 5F). However, a increase in retinal apoptosis was noted in the SOR/IR820 NPs plus light irradiation group, ≈2.2 times higher than the SOR/IR820‐CA4 NPs plus light irradiation group. These findings suggest that the limited PTT/PDT properties of IR820‐CA4 prodrug contribute to its enhanced ocular biosafety.

## 3 Discussion

Effective drug delivery to the posterior segment of the eyes via non‐invasive methods remains challenging.^[^ ^44^ ^]^ Owing to the intrinsic light‐adapted nature of the eyes, it would be of great interest to explore photoresponsive nanoplatforms for ocular drug delivery. Previous research by our laboratory has shown that it is feasible to use light‐triggered drug release to effectively accumulate drugs in ocular lesions.^[^ ^45^, ^46^ ^]^ PDT is the clinically available angio‐occlusive method, but it can induce some unfavorable secondary responses such as angiogenesis, which undermines the therapeutic efficacy of vascular occlusion.^[^ ^47^, ^48^ ^]^ As a potential alternative, vascular disrupting agents can induce CNV vessel regression. However, systemic exposure to vascular disrupting drugs is associated with adverse vascular events. Herein, taking advantage of the photodegradation mechanism of heptamethine cyanine upon NIR light irradiation, we employed the prodrug strategy for the design of a photocaged prodrug IR820‐CA4. Interestingly, we observed limited PTT/PDT properties of IR820‐CA4 under 690 nm light irradiation, which would help minimize the risks of hyperthermia and oxidative stress when extending the light irradiation time.

In this study, we presented an approach for delivering dual‐function anti‐vascular drugs to CNV lesions through intravenous injection of photoactivatable nanoparticles (SOR/IR820‐CA4 NPs). The formed SOR/IR820‐CA4 NPs possess ultrahigh drug loading capacity (97.1% and 2.9% for SOR and CA4, respectively), good colloidal stability, and NIR light‐sensitive drug release. In this way, controlled release of anti‐angiogenic agent SOR and vascular disrupting agent CA4 in the ocular tissue can be achieved by intravenous administration of SOR/IR820‐CA4 NPs and light illumination to the eyes. Consequently, this strategy achieved remarkable downregulation of angiogenic signaling, alleviated neovascular leakage, and gave rise to an ≈87% suppression of CNV growth in a laser‐induced CNV mouse model. Moreover, SOR/IR820‐CA4 NP treatment did not cause noticeable systemic and localized side effects, mainly due to the inactivity of IR820‐CA4 prodrug in the dark and minimal photo‐damaging effects upon light irradiation. This study presents a promising paradigm to overcome the limitations of conventional intravitreal administration through intravenous treatment of CNV using light‐activatable anti‐vascular agents. This treatment method also has extensive potential applications in treating tumors and various other neovascular eye diseases, including retinal vein occlusions and diabetic retinopathy.

## 4 Experimental Section

### Materials

Combretastatin A4, sorafenib, 4‐Nitrophenyl chloroformate, N‐ethyl diisopropylamine, and *N,N*'‐dimethylethylenediamine were obtained from Macklin Biochemical Co., Ltd (Shanghai, China). IR820 dye was purchased from Bidepharm Co., Ltd. (Shanghai, China). Singlet Oxygen Sensor Green (SOSG), 3‐(4, 5‐dimethylthiazol‐2‐yl) 2, 5‐diphenyl tetrazolium bromide (MTT), and Pierce BCA Protein Assay Kit were purchased from Thermo Fisher Scientific, Inc. (Eugene, USA). Matrigel matrix (356234) was obtained from Corning, Inc. (New York, USA). Human umbilical vein endothelial cells (HUVECs) and human retinal pigment epithelial cells (ARPE‐19) were purchased from the American Type Culture Collection (Manassas, VA, USA). All chemicals used were analytical or ACS reagent grade.

### Synthesis of IR820‐CA4

R1: Combretastatin A‐4 (CA4, 31.6 mg, 0.1 mmol, 1 eq.) and 4‐Nitrophenyl chloroformate (60.5 mg, 0.3 mmol, 3 eq.) were dissolved in 1 mL anhydrous DCM in a dry, nitrogen‐filled flask. The mixture was stirred at room temperature overnight. The reaction was monitored by thin‐layer chromatography to confirm the complete consumption of CA4 without further purification.

R2: IR820 (127.4 mg, 0.15 mmol, 1.5 eq.) was dissolved in 1 mL anhydrous DMF in a dry, nitrogen‐filled flask. Then the mixture of N‐ethyl diisopropylamine (DIPEA, 0.15 mmol, 25.8 µL, 1.5 eq.) and *N,N*'‐dimethylethylenediamine (DMA, 0.3 mmol, 29.4 µL, 3 eq.) was added into the solution. The mixture was stirred at 70 °C for ≈5 min and then immediately dripped into 200 mL cold ethyl ether. The precipitation was collected by centrifugation (2000 g, 10 min) at 4 °C and dried in a vacuum desiccator. The consumption of IR820 was confirmed by high‐performance liquid chromatography (HPLC) without further purification.

The crude product of R2 was dissolved in 1 mL of anhydrous DMF in a dry, nitrogen‐filled flask. Then the solution of R1 was slowly dropped into the reaction mixture. The mixed solution was stirred for 24 h, followed by purification with HPLC and freeze‐drying. The purified product was confirmed by proton nuclear magnetic resonance and mass spectroscopy. IR820‐CA4 lyophilized powder was stored at ‐20 °C protected from light.

### Photolysis of IR820‐CA4 and CA4 Release

To investigate the photolysis efficiency of IR820‐CA4, the aqueous solution of 20 µg mL^−1^ IR820‐CA4 was exposed to 690 nm laser irradiation (80 mW cm^−2^) for different time intervals (0, 1, 2, 3, 5, and 8 min). Subsequently, IR820‐CA4 solution was injected into the HPLC system for analyzing the remaining prodrug. Meanwhile, UV/–Vis absorption and fluorescence spectra of IR820‐CA4 were recorded by SpectraMax M4 multi‐mode microplate reader before and after irradiation.

To characterize NIR light‐triggered CA4 release kinetics, 690 nm laser irradiation (80 mW cm^−2^, 8 min) was applied to the aqueous solution of IR820‐CA4. Then IR820‐CA4 solution was incubated in a shaking water bath at 37 °C, 120 rpm. The solution was collected at selected time points (0, 1, 2, 4,16, 24, 36, and 48 h) and analyzed by HPLC for detecting the released CA4.

### Fabrication and Characterization of SOR/IR820‐CA4 NPs

SOR/IR820‐CA4 NPs were obtained by an optimized nanoprecipitation method. Briefly, 0.4 mg SOR and 0.02 mg IR820‐CA4 were co‐dissolved in 5 µL DMSO. The mixture solution was quickly dripped into 0.4 mL of stirring water under vortex, followed by centrifugation at 4000 g for 5 min to remove the precipitant. Thereafter, SOR/IR820‐CA4 NPs were readily collected by centrifugation at 3 × 10^4^ g for 15 min and redispersed in distilled water. Meanwhile, the nanoparticles (SOR/IR820 NPs) prepared with the same feeding ratio of SOR and IR820 served as the control.

For nanoparticle characterization, the polydispersity index (PDI) and surface potential of SOR/IR820‐CA4 NPs were measured by Zetasizer Nano ZS (Malvern, UK). The morphology of the nanoparticles was observed under a Philips CM100 transmission electron microscope. UV–Vis absorption spectrum of SOR/IR820‐CA4 NPs was acquired by a multi‐mode microplate reader. Besides, the encapsulation efficiency and drug‐loading capacity of SOR/IR820‐CA4 NPs were quantified by HPLC and calculated according to the previous report.^[^ ^49^ ^]^

### In Vitro Photothermal Effect and Single Oxygen Generation by SOR/IR820‐CA4 NPs

To assess the photothermal effect of SOR/IR820‐CA4 NPs, SOR/IR820‐CA4 NP and SOR/IR820 NP solutions were placed into 1.5 mL Eppendorf tubes at the room temperature, and irradiated with a 690 nm laser (80 mW cm^−2^) for varying periods (0, 1, 3, 5, and 8 min). The temperature variations of the nanoparticle solutions were monitored by the FLIR E54 thermal imaging camera. The control experiments were performed with the predetermined procedure as above with the same volume of distilled water.

In addition, Singlet Oxygen Sensor Green (SOSG) was used as a probe to determine the in vitro ROS generation. Briefly, SOSG was added into SOR/IR820‐CA4 NP or SOR/IR820 NP solutions to the equivalent concentrations of 5 µm IR820‐CA4 and 1 µm SOSG. Next, a 690 nm laser (80 mW cm^−2^) was used to illuminate the mixture solutions for varying periods (0, 1 min, 3 min, 5 min, 8 min, 10 min). The fluorescence intensity of SOSG was detected by a microplate reader with the excitation/emission wavelength of 504/525 nm. SOSG solution exposed to the same irradiation dose was used as the control.

### In Vitro SOR Release

NIR‐light‐triggered release of SOR was conducted by the dialysis method. SOR/IR820‐CA4 NP or SOR/IR820 NP solutions were added into dialysis sacks with a molecular‐weight cut‐off (MWCO) pore size of 2 × 10^4^ Da. The drug‐contained dialysis sacks were immersed in 8 mL PBS and incubated in a shaking water bath at 37 °C, 120 rpm. At predetermined time points (0, 10 min, 30 min, 1 h, 1 h 10 min, 1 h 30 min, 2, 4, 8, 12, 24, and 48 h), release media were immediately replaced with 8 mL solution of fresh PBS. 690 nm laser irradiation was applied to the nanoparticle solutions at 1 h post‐incubation (80 mW cm^−2^, 5 min). To ensure the dialysis setting maintains the near‐infinite sink conditions, free SOR solution was used as the control. SOR content in release media was analyzed by HPLC analysis.

### In Vitro Cell Uptake of SOR/IR820‐CA4 NPs

Human umbilical vein endothelial cells (HUVECs) were seeded in a 24‐well plate and cultured overnight. Then cells were incubated with different formulations at the equivalent concentration of 0.2 µm IR820‐CA4 at 37 °C for 4 h. Later, cells were rinsed with PBS for three times and collected for flow cytometry analysis using Agilent Novocyte Advanteon BVR cytometer (Ex/Em = ≈640 nm/780 nm).

### Angiogenesis Assays

The effects of SOR/IR820‐CA4 NPs on angiogenic activities of HUVECs were studied by transwell migration assay and tube formation assay. For transwell migration assay, HUVEC suspensions were seeded into the transwell insert (8 µm of pore size) in DMEM basal media containing 20 ng mL^−1^ VEGF_165_ and different formulations at the equivalent concentration of 2 µm SOR. Meanwhile, 650 µL of DMEM media containing 10% FBS was added to each well of 24‐well plates. Then the plates with transwell were exposed to 690 nm light irradiation at 4 h post‐incubation (80 mW cm^−2^, 5 min) and cultured for another 12 h at 37 °C. Cells attached to the upper compartment of the transwell were gently wiped off with the cotton swab, while the migrated cells on the bottom of the transwell were stained with 1% crystal violet (w/v) and imaged with Olympus DP74 inverted microscope. The migrated cell area per field was quantified by Image J.

For tube formation assay, HUVECs were suspended in DEME basal media containing 20 ng mL^−1^ VEGF_165_ and various formulations at the equivalent concentration of 2 µm SOR and then plated on a Matrigel‐coated 96‐well plate. Cells were irradiated with 690 nm laser irradiation immediately or not (80 mW cm^−2^, 5 min). Images of tube formation trend were taken at 4 h post‐incubation. The number of meshes and total branching length of tubular structures were subsequently analyzed by Image J.

### Visualization of Microtubule Depolymerization

HUVECs were seeded on 35 mm‐diameter confocal dishes and grown to 70% of confluency. After that, cells were treated with various formulations at the equivalent concentration of 10 nm IR820‐CA4 at 37 °C for 4 h, followed by light irradiation (690 nm, 80 mW cm^−2^, 5 min) or not. After 12 h of incubation, the culture media were removed and cells were gently washed with PBS buffer for three times. Cells were fixed with 4% paraformaldehyde for 20 min, washed with PBS buffer, permeabilized in 0.1% Triton X‐100, and then stained with Alexa Fluor 488‐conjugated phalloidin (1:400 dilution, A12379, ThermoFisher) and DAPI buffer (1:1 × 10^4^, D1306, ThermoFisher) for 30 min according to the manufacturer's recommendations. The dishes were observed under the ZEISS LSM 900 confocal microscope.

### In Vitro Cytotoxicity Study

HUVECs or human retinal pigment epithelial cells ARPE‐19 were seeded into 96‐well plates and grown in a cell culture incubator at 37 °C overnight. Then cells were exposed to fresh cell culture media containing different formulations, followed by 690 nm laser irradiation (80 mW cm^−2^, 5 min) or not. The cells were continuously incubated for 48 h. Afterward, 10 µL of 10 mg mL^−1^ MTT reagent (3‐(4,5‐dimethylthiazol‐2‐yl)−2,5‐diphenyltetrazolium bromide) was added to each well and additionally incubated at 37 °C for 4 h. The culture medium was replaced with 200 µL of DMSO to solubilize the generated formazan crystals. The optical density of each well was determined by a microplate reader at the wavelength of 570 nm. Cell viability data were normalized to the untreated cells.

### Pharmacokinetics and Intraocular Drug Distribution

C57BL/6 mice (20–25 g, 6–8 weeks) were intravenously administrated with different formulations (SOR, IR820‐CA4, or SOR/IR820‐CA4 NPs) at the equivalent dose of 10 mg kg^−1^ SOR. ≈25 µL of blood samples were collected in heparinized tubes at different time points (5, 30 min, 1, 3, 6, and 12 h) and subject to centrifugation (2000 g, 10 min, 4 °C) for plasma separation from the whole blood samples. To study the intraocular drug distribution of formulations, mice were sacrificed at 1 h post‐intravenous injection. The choroid‐RPE, retina, and other ocular tissue of mice were surgically excised and homogenized at 4 °C. Then 10 µL of plasma samples or tissue homogenate were thoroughly mixed with 30 µL of acetonitrile, followed by ultra‐centrifugation (2 × 10^4 ^g, 20 min, 4 °C). The drug concentrations of the supernatant were detected by HPLC analysis. Plasma drug concentration data were analyzed using PKSolver with noncompartmental data analysis mode.

### Construction of CNV Mouse Model and Animal Treatment

Laser‐induced CNV mouse model was established following the standard protocols as previously reported.^[^ ^50^, ^51^, ^52^, ^53^ ^]^ Briefly, C57BL/6 mice (20–25 g, 6–8 weeks) were anesthetized by intraperitoneal injection of the mixture solution of ketamine (100 mg kg^−1^) and xylazine (10 mg kg^−1^). Then the mice were instilled with 1% tropicamide eye drops for pupil dilation, and then administered with 0.5% tetracaine eye drops and carboxymethylcellulose topical drops for topical anesthesia and hydration. Four laser pulses were applied to the posterior pole of the mouse eye with the OcuLight Infrared Laser System (75 µm spot size, 75 ms duration, 120 mW) at ≈3, 6, 9, and 12 o'clock positions. The generated vaporization bubble demonstrated successful photocoagulation of the Bruch's membrane.

Subsequently, CNV mice were randomized to receive the following treatment regimens: 1) Saline; 2) SOR solution; 3) IR820‐CA4 solution plus light irradiation; 4) SOR/IR820‐CA4 NPs; 5) SOR/IR820 NPs plus light irradiation; 6) SOR/IR820‐CA4 NPs plus light irradiation; 7) Saline plus light irradiation. The mice were treated with the aforementioned formulations at the equivalent dose of 10 mg kg^−1^ SOR via tail vein injection on day 3 and day 5, respectively. After drug administration, the mouse eyes were immediately administered with 1% tropicamide eyedrop, 0.5% tetracaine eye drops, and carboxymethylcellulose topical drops for pupil dilation, topical anesthesia, and lubrication. Light irradiation procedures were performed with a 690 nm laser diode laser (80 mW cm^−2^, 5 min). The mice were sacrificed on day 7 for therapeutic and biosafety evaluations. All animal study procedures were approved by the Institutional Animal Care and Use Committee (IACUC), Zhongshan Ophthalmic Center, Sun Yat‐sen University (IACUC no. Z2022059).

### Optical Coherence Tomography Angiography

On day 7, the regression of CNV lesions was examined by Heidelberg Spectralis optical coherence tomography (OCT) system. The CNV lesions were identified as spindle/dome‐shaped hyperreflective complexes above the RPE layer. The cross–sectional OCT angiograms were obtained by vertical scans through the center of CNV lesions.

### Fundus Fluorescein Angiography

Vascular leakage from CNV lesions was examined by fundus fluorescein angiography (FFA) on day 7. CNV mice were anesthetized and intraperitoneally administrated with 0.2 mL of 2% fluorescein sodium. Fluorescein angiograms were recorded at ≈5 min post‐fluorescein administration by Micron IV imaging system. Lesion leakage severity was graded by masked researchers according to the established criteria^[^ ^54^ ^]^: Grade I: No hyperfluorescence; Grade II: Hyperfluorescence without leakage; Grade III: Hyperfluorescence and late mild leakage; Grade IV: Bright hyperfluorescence with severe fluorescein leakage.

### Western Blotting Assay

Cell samples or RPE‐choroid homogenate were lysed with RIPA buffer containing 1% phosphatase and protease inhibitor cocktails. The total protein concentrations were then determined by the bicinchoninic acid assay kit (23225, ThermoFisher) according to the manufacturer's instructions. The normalized protein samples were mixed with 5 × sodium dodecyl sulfate (SDS) sample loading buffer (P0015, Beyotime) and denatured at 100 °C for 10 min. Western blot assay was conducted using a Bio‐Rad SDS electrophoresis system according to the manufacturer's protocols. After being blocked by 1× Tris‐buffered saline containing 0.1% Tween 20 (TBST) and 5% bovine serum albumin, the membranes were incubated with the primary antibodies at 4 °C overnight. After being washed with TBST buffer for three times and incubated with horseradish peroxidase (HPR)‐conjugated secondary antibodies for 1 h, the blots were detected by BeyoECL Plus chemiluminescent substrate (P0018S, Beyotime) and imaged by Bio‐Rad ChemiDoc Imaging system. The used antibodies were listed as followed: VEGFR2 antibody (1:500 dilution, A5609, ABclonal), Phospho‐VEGFR2 (Tyr1054/Tyr1059) antibody (1:500 dilution, ab5473, Abcam), ERK1/2 antibody (1:1000 dilution, A16686, ABclonal), Phospho‐ERK1 (T202/Y204) + ERK2 (T185/Y187) antibody (1:500 dilution, AP0472, ABclonal), MEK1/2 antibody (1:1000 dilution, A4868, ABclonal), Phospho‐MEK1/2 (Ser217/221) antibody (1:500 dilution, AP134, ABclonal), GAPDH antibody (1:1000 dilution, AC001, ABclonal), CD31 antibody (1:500 dilution, GB11063, Servicebio), and Goat Anti‐Rabbit IgG H&L (HRP) antibody (1:2000 dilution, ab6721, Abcam).

### Immunofluorescent Staining of CD31 and Ki67

To quantitatively analyze the CNV area and the percentage of proliferating cells, the mouse eyeballs were fixed in 4% paraformaldehyde buffer and then enucleated for the preparation of RPE‐choroid complexes. After being blocked and permeabilized, RPE‐choroid complexes were co‐incubated with CD31 antibody (1:200 dilution, MAB1398Z, Millipore) and Ki67 antibody (1:200 dilution, 9129S, Cell Signaling) at 4 °C overnight. The tissue was washed with PBS buffer and then incubated with Goat Anti‐Hamster IgG H&L (Alexa Fluor 488) antibody (1:1000 dilution, ab173003, Abcam), Goat Anti‐Rabbit IgG H&L (Alexa Fluor 647) antibody (1:1000 dilution, ab150079, Abcam) and DAPI buffer (1:10 000, D1306, ThermoFisher) at the room temperature for 2 h. After being washed with PBS buffer, the tissue was flat‐mounted on the glass sides with four incisions made and observed under ZEISS LSM 980 confocal microscope.

### Immunohistochemical Assay

Paraffin‐embedded eyeballs were cut into 10 µm cross–sections in a fashion perpendicular to the optic disc while parallel to the optical axis. After dewaxing in xylene solutions and rehydration in gradient ethanol solutions, the slides were stained with hematoxylin&eosin (H&E) and reviewed by Olympus DP74 inverted microscope.

### Statistical Analysis

All the data were expressed as mean and standard deviation (SD) except for specific notifications. The statistical analysis was done by either unpaired two‐tailed student's t‐test (between‐group analysis) or a one‐way ANOVA test combined with Dunnett's post hoc test (multi‐group analysis). Clinical grading results of fluorescein leakage were analyzed with Chi‐square tests. *p* < 0.05 was considered as statistical significance.

## Conflict of Interest

The authors declare no conflict of interest.

## Author Contributions

S.X. and J.L. contributed equally to this work; S. X. and W.W. conceived the study; S. X. carried out the experiments with the help of J. L. and K.L. under the supervision of W. W. and X. L.; S. X. wrote the first draft of the manuscript; All authors reviewed, discussed the manuscript, and provided approval to the final version.

## Supporting information

Supporting Information

## Data Availability

The data that support the findings of this study are available from the corresponding author upon reasonable request.

## References

[1] Y. Yanagi , V. H. X. Foo , A. Yoshida , Eye (Lond) 2019, 33, 34. 30315261 10.1038/s41433-018-0225-x PMC6328602

[2] W. R. Green , D. Wilson , J. Ophthalmology 1986, 93, 1169. 10.1016/s0161-6420(86)33609-1 2433662

[3] M. Fleckenstein , T. D. L. Keenan , R. H. Guymer , U. Chakravarthy , S. Schmitz‐Valckenberg , C. C. Klaver , W. T. Wong , E. Y. Chew , Nat. Rev. Dis. Primers 2021, 7, 31. 33958600 10.1038/s41572-021-00265-2 PMC12878645

[4] A. Sarkar , S. Dyawanapelly , J Control Release 2021, 329, 1262. 33129920 10.1016/j.jconrel.2020.10.054

[5] W. L. Wong , X. Su , X. Li , C. M. G. Cheung , R. Klein , C.‐Y. Cheng , T. Y. Wong , Lancet Glob Health 2014, 2, 106. 10.1016/S2214-109X(13)70145-1 25104651

[6] Y. Wu , X. Li , X. Fu , X. Huang , S. Zhang , N. Zhao , X. Ma , Q. Saiding , M. Yang , W. Tao , X. Zhou , J. Huang , Adv. Sci. (Weinh) 2024, e2403399. 39031809 10.1002/advs.202403399 PMC11348104

[7] Q. Qi , Y. Wei , X. Zhang , J. Guan , S. Mao , J Control Release 2023, 361, 191. 37532148 10.1016/j.jconrel.2023.07.055

[8] K. G. Falavarjani , Q. D. Nguyen , Eye (Lond) 2013, 27, 787. 23722722 10.1038/eye.2013.107 PMC3709385

[9] J. Bradley , M. Ju , G. S. Robinson , Angiogenesis 2007, 10, 141. 17372853 10.1007/s10456-007-9069-x

[10] T. H. Lim , T. Y. Y. Lai , K. Takahashi , T. Y. Wong , L. J. Chen , P. Ruamviboonsuk , C. S. Tan , W. K.i Lee , C. M. G. Cheung , N. F. Ngah , R. Patalauskaite , P. Margaron , A. Koh , JAMA Ophthalmol 2020, 138, 935. 32672800 10.1001/jamaophthalmol.2020.2443 PMC7366282

[11] A. C. Dudley , A. W. Griffioen , Angiogenesis 2023, 26, 313. 37060495 10.1007/s10456-023-09876-7 PMC10105163

[12] H. Nambu , R. Nambu , M. Melia , P. A. Campochiaro , Invest Ophthalmol Vis Sci 2003, 44, 3650. 12882819 10.1167/iovs.02-0985

[13] K. Zhang , L. Zhang , R. N. Weinreb , Nat Rev Drug Discov 2012, 11, 541. 22699774 10.1038/nrd3745

[14] C. Dumontet , M. A. Jordan , Nat Rev Drug Discov 2010, 9, 790. 20885410 10.1038/nrd3253 PMC3194401

[15] M. Karimi , A. Ghasemi , P. Sahandi Zangabad , R. Rahighi , S. M. Moosavi Basri , H. Mirshekari , M. Amiri , Z. Shafaei Pishabad , A. Aslani , M. Bozorgomid , D. Ghosh , A. Beyzavi , A. Vaseghi , A. R. Aref , L. Haghani , S. Bahrami , M. R. Hamblin , Chem. Soc. Rev. 2016, 45, 1457. 26776487 10.1039/c5cs00798d PMC4775468

[16] Y. Xue , H. Bai , B. Peng , B. Fang , J. Baell , L. Li , W. Huang , N. H. Voelcker , Chem. Soc. Rev. 2021, 50, 4872. 33734247 10.1039/d0cs01061h

[17] X. Lin , X. Wu , X. Chen , B. Wang , W. Xu , Int. J. Pharm. 2021, 602, 120591. 33845152 10.1016/j.ijpharm.2021.120591

[18] X. Wang , F. Luan , H. Yue , C. Song , S. Wang , J. Feng , X. Zhang , W. Yang , Y. Li , W. Wei , Y. Tao , Adv Drug Deliv Rev 2023, 200, 115006. 37451500 10.1016/j.addr.2023.115006

[19] J. Liu , W. Kang , W. Wang , Photochem. Photobiol. 2022, 98, 288. 34861053 10.1111/php.13570

[20] A. Y. Rwei , W. Wang , D. S. Kohane , Nano Today 2015, 10, 451. 26644797 10.1016/j.nantod.2015.06.004 PMC4669578

[21] Y. Wang , C. H. Liu , T. Ji , M. Mehta , W. Wang , E. Marino , J. Chen , D. S. Kohane , Nat. Commun. 2019, 10, 804. 30778060 10.1038/s41467-019-08690-4 PMC6379485

[22] K. Long , Y. Wang , W. Lv , Y. Yang , S. Xu , C. Zhan , W. Wang , Bioeng. Transl. Med. 2022, 7, e10311. 36176605 10.1002/btm2.10311 PMC9472000

[23] Y. Li , W. Lv , L. Wang , Y. Zhang , L. Yang , T. Wang , L. Zhu , Y. Wang , W. Wang , Nano Res. 2021, 14, 2630.

[24] K. Long , Y. Yang , W. Lv , K. Jiang , Y. Li , A. C. Y. Lo , W. C. Lam , C. Zhan , W. Wang , Adv. Sci. 2021, 8, e2101754. 10.1002/advs.202101754 PMC8529428 34448360

[25] H. Janekova , M. Russo , U. Ziegler , P. Stacko , Angew Chem Int Ed Engl 2022, 61, e202204391. 35578980 10.1002/anie.202204391 PMC9542589

[26] R. Tapia Hernandez , M. C. Lee , A. K. Yadav , J. Chan , J. Am. Chem. Soc. 2022, 144, 18101. 36153991 10.1021/jacs.2c08187 PMC10088867

[27] R. Weinstain , T. Slanina , D. Kand , P. Klan , Chem. Rev. 2020, 120, 13135. 33125209 10.1021/acs.chemrev.0c00663 PMC7833475

[28] Y. Li , Y. Zhou , X. Yue , Z. C. Dai Bioact Mater 2021, 6, 794. 33024900 10.1016/j.bioactmat.2020.09.009 PMC7528000

[29] Y. Yang , K. Long , Y. Wang , L. Li , J. Shi , J. Liu , L. Kong , L. Yu , J. Ding , Z. Huang , W. Wang , C. Zhan , Adv. Healthcare Mater. 2022, 11, 2102362. 10.1002/adhm.202102362 34851048

[30] R. R. Nani , A. P. Gorka , T. Nagaya , H. Kobayashi , M. J. Schnermann , Angew Chem Int Ed Engl 2015, 54, 13635. 26403799 10.1002/anie.201507391 PMC4743669

[31] L.u Lu , K. Wang , C. Lin , W. Yang , Q. Duan , K.e Li , K. Cai , Biomaterials 2021, 279, 121193. 34700227 10.1016/j.biomaterials.2021.121193

[32] M. M. Leitao , D. de Melo‐Diogo , C. G. Alves , R. Lima‐Sousa , I. J. Correia , Adv. Healthcare Mater. 2020, 9, 1901665. 10.1002/adhm.201901665 31994354

[33] A. P. Gorka , M. J. Schnermann , Curr. Opin. Chem. Biol. 2016, 33, 117. 27348157 10.1016/j.cbpa.2016.05.022 PMC7383357

[34] A. F. Moleiro , G. Conceicao , A. F. Leite‐Moreira , A. Rocha‐Sousa , J Ophthalmol 2017, 2017, 3034953. 28848677 10.1155/2017/3034953 PMC5564124

[35] H. Yang , H. Mao , Z. Wan , A. Zhu , M. Guo , Y. Li , X. Li , J. Wan , X. Yang , X. Shuai , H. Chen , Biomaterials 2013, 34, 9124. 24008037 10.1016/j.biomaterials.2013.08.022

[36] L. Liu , Y. Cao , C. Chen , X. Zhang , A. McNabola , D. Wilkie , S. Wilhelm , M. Lynch , C. Carter , Cancer Res. 2006, 66, 11851. 17178882 10.1158/0008-5472.CAN-06-1377

[37] M. Simons , E. Gordon , L. Claesson‐Welsh , Nat. Rev. Mol. Cell Biol. 2016, 17, 611. 27461391 10.1038/nrm.2016.87

[38] G. M. Tozer , C. Kanthou , B. C. Baguley , Nat. Rev. Cancer 2005, 5, 423. 15928673 10.1038/nrc1628

[39] P. Bhattarai , Z. Dai , Adv. Healthcare Mater. 2017, 6, 1700262. 10.1002/adhm.201700262 28558146

[40] R. Hennig , A. Ohlmann , J. Staffel , K. Pollinger , A. Haunberger , M. Breunig , F. Schweda , E. R. Tamm , A. Goepferich , J Control Release 2015, 220, 265. 26494258 10.1016/j.jconrel.2015.10.033

[41] R. Hennig , A. Goepferich , Eur. J. Pharm. Biopharm. 2015, 95, 294. 25758124 10.1016/j.ejpb.2015.02.027

[42] Y. Xu , K. Cui , J. Li , X. Tang , J. Lin , X.i Lu , R. Huang , B. Yang , Y. Shi , D. Ye , J. Huang , S. Yu , X. Liang , J. Pineal Res. 2020, 69, 12660. 10.1111/jpi.12660 32323368

[43] P. Urban , N. J. Liptrott , S. Bremer , Wiley Interdiscip Rev Nanomed Nanobiotechnol 2019, 11, 1546. 10.1002/wnan.1546 PMC7816241 30556649

[44] F. J. Cabrera , D. C. Wang , K. Reddy , G. Acharya , C. S. Shin , Drug Discov Today 2019, 24, 1679. 31175955 10.1016/j.drudis.2019.05.035 PMC6708448

[45] K. Long , Y. Yang , W. Lv , K. Jiang , Y. Li , A. C. Y. Lo , W. C. Lam , C. Zhan , W. Wang , Adv. Sci. 2021, 8, 2101754. 10.1002/advs.202101754 PMC8529428 34448360

[46] S. Xu , K. Cui , K. Long , J. Li , N.i Fan , W. C. Lam , X. Liang , W. Wang , Adv. Sci. 2023, 10, 2301985. 10.1002/advs.202301985 PMC10625062 37705491

[47] M. F. Zuluaga , C. Mailhos , G. Robinson , D. T. Shima , R. Gurny , N. Lange , Invest Ophthalmol Vis Sci 2007, 48, 1767. 17389510 10.1167/iovs.06-1224

[48] U. Schmidt‐Erfurth , U. Schlo¨tzer‐Schrehard , C. Cursiefen , S. Michels , A. Beckendorf , G. O. H. Naumann , Invest Ophthalmol Vis Sci 2003, 44, 4473. 14507895 10.1167/iovs.02-1115

[49] K. Cai , X.i He , Z. Song , Q. Yin , Y. Zhang , F. M. Uckun , C. Jiang , J. Cheng , J. Am. Chem. Soc. 2015, 137, 3458. 25741752 10.1021/ja513034e

[50] V. Lambert , J. Lecomte , S. Hansen , S. Blacher , M.‐L. A. Gonzalez , I. Struman , N. E. Sounni , E. Rozet , P. de Tullio , J. M. Foidart , J. M. Rakic , A. Noel , Nat. Protoc. 2013, 8, 2197. 24136346 10.1038/nprot.2013.135

[51] S. Yang , T. Li , H. Jia , M. Gao , Y. Li , X. Wan , Z. Huang , M. Li , Y. Zhai , X. Li , X. Yang , T. Wang , J. Liang , Q. Gu , X. Luo , L. Qian , S. Lu , J. Liu , Y. Song , F. Wang , X. Sun , D. Yu , Sci. Transl. Med. 2022, 14, eabj2177. 35648811 10.1126/scitranslmed.abj2177

[52] H. M. Zhang , X. H. Li , M. Chen , J. Luo , Int J Ophthalmol 2020, 13, 886. 32566498 10.18240/ijo.2020.06.05 PMC7270251

[53] X. Liu , A. Guo , Y. Tu , W. Li , L. Li , W. Liu , Y. Ju , Y. Zhou , A. Sang , M. Zhu , Cell Death Dis. 2020, 11, 1016. 33247124 10.1038/s41419-020-03222-1 PMC7695853

[54] J. Shen , H. Gao , L. Chen , Y. Jiang , S. Li , Y.u Chao , N. Liu , Y. Wang , T. Wei , Y. Liu , J. Li , M. Chen , J. Zhu , J. Liang , X. Zhou , X. Zhang , P. Gu , Q. Chen , Z. Liu , Sci. Adv. 2023, 9, eabq3104. 36706184 10.1126/sciadv.abq3104 PMC9882978

