# Correction to “Light‐Activated Anti‐Vascular Combination Therapy against Choroidal Neovascularization”

**DOI:** 10.1002/advs.202416443

**Published:** 2025-01-13

Xu S, Li J, Long K, Liang X, Wang W. Light‐Activated Anti‐Vascular Combination Therapy against Choroidal Neovascularization. *Advanced Science* **2024**, 11(40), 2404218.

https://doi.org/10.1002/advs.202404218

In Figure 3A, the “Control + hv” image was mistakenly chosen from the group of “Control” during the layout of the images. As the quantification analysis of the migrated cell area (Figure 3B) was based on the correct images, the results and conclusions remain unchanged.


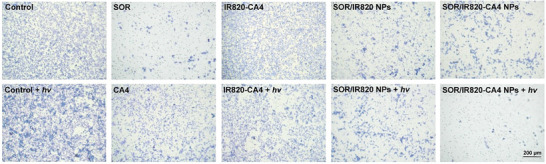


We apologize for this error.

