# Supplementary material for: Light‐Activated Anti‐Vascular Combination Therapy against Choroidal Neovascularization
Source: Adv Sci (Weinh). 2024 Aug 29;11(40):2404218. doi: 10.1002/advs.202404218 (PMC11516295; doi:10.1002/advs.202404218)
Supplement: Supplementary file 1 — Supporting Information [file ADVS-11-2404218-s001.docx]

**Supporting information**

**Light-activated anti-vascular combination therapy against choroidal neovascularization**

**Shuting Xu†^1, 2, 3^, Jia Li†^1, 2, 3^, Kaiqi Long^1, 2, 3^, Xiaoling Liang*^4^, Weiping Wang*^1, 2, 3^**

1 State Key Laboratory of Pharmaceutical Biotechnology, The University of Hong Kong, Hong Kong, China

2 Department of Pharmacology and Pharmacy, Li Ka Shing Faculty of Medicine, The University of Hong Kong, Hong Kong, China.

3 Laboratory of Molecular Engineering and Nanomedicine, Dr. Li Dak-Sum Research Centre, The University of Hong Kong, Hong Kong, China.

4 State Key Laboratory of Ophthalmology, Zhongshan Ophthalmic Center, Sun Yat-sen University, Guangdong Provincial Key Laboratory of Ophthalmology and Visual Science, Guangzhou 510060, China.

† These authors made equal contributions to this work

*Corresponding author, Prof. X. Liang, E-mail address: [liangxl2@mail.sysu.edu.cn](mailto:liangxl2@mail.sysu.edu.cn); Dr. W. Wang, E-mail address: [wangwp@hku.hk](mailto:wangwp@hku.hk)

**
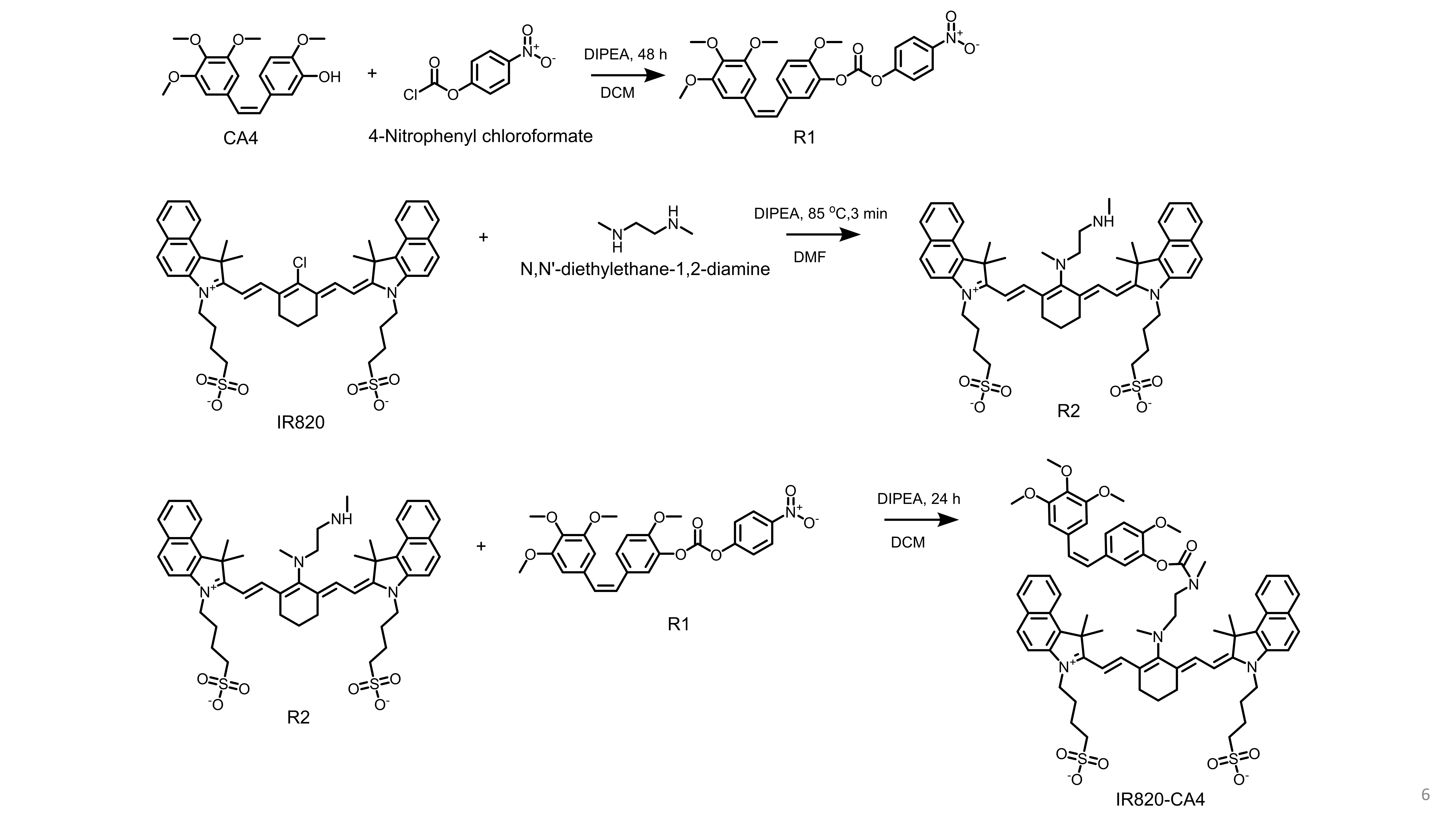
Reshults**

Figure S1. Synthesis route of IR820-CA4.


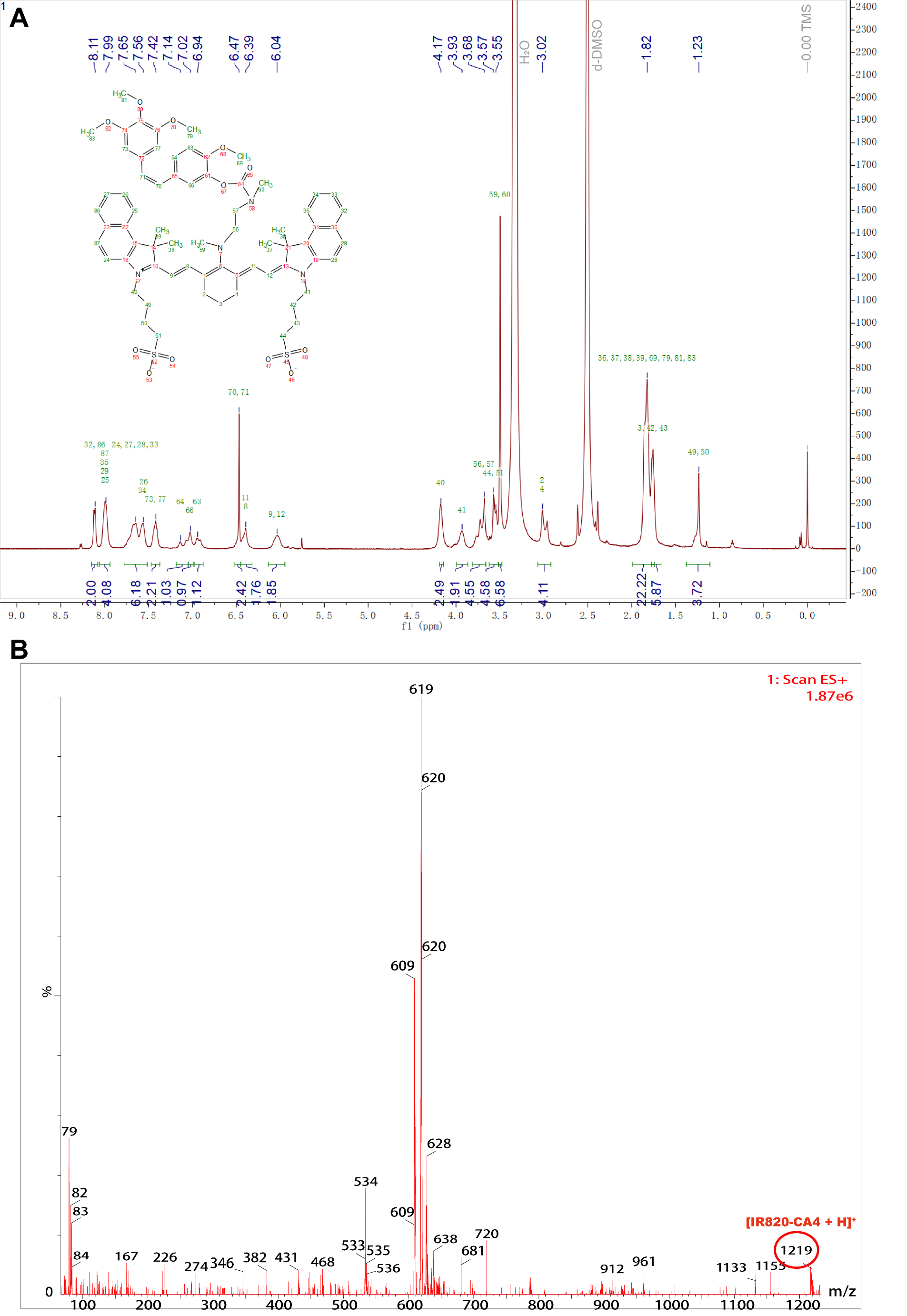
Figure S2. (A) Proton nuclear magnetic resonance spectrum and (B) mass spectrum of IR820-CA4.


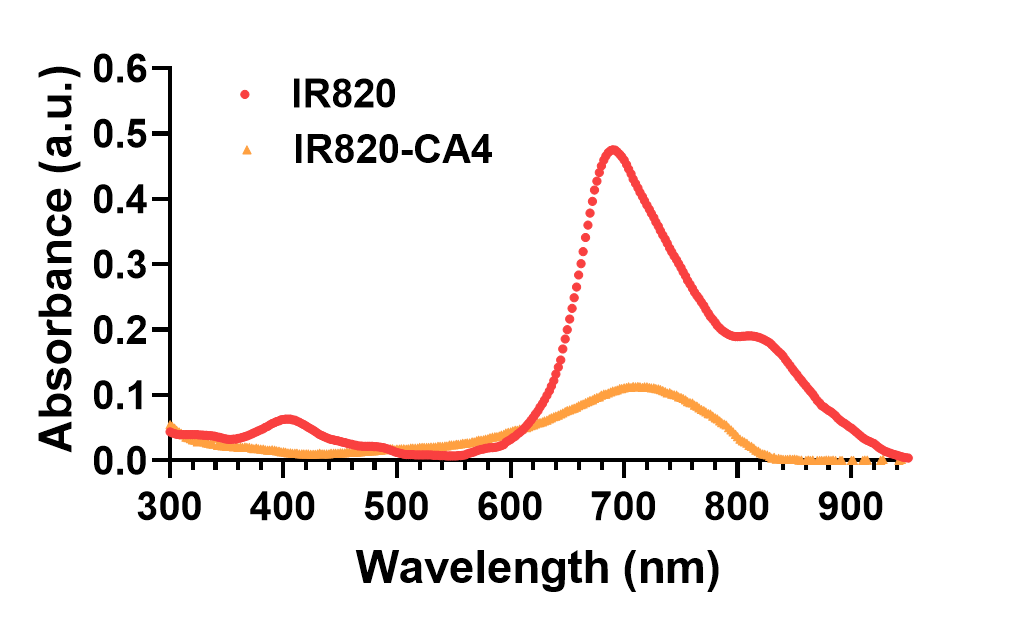
Figure S3. Representative absorption spectra of IR820 and IR820-CA4 in water.


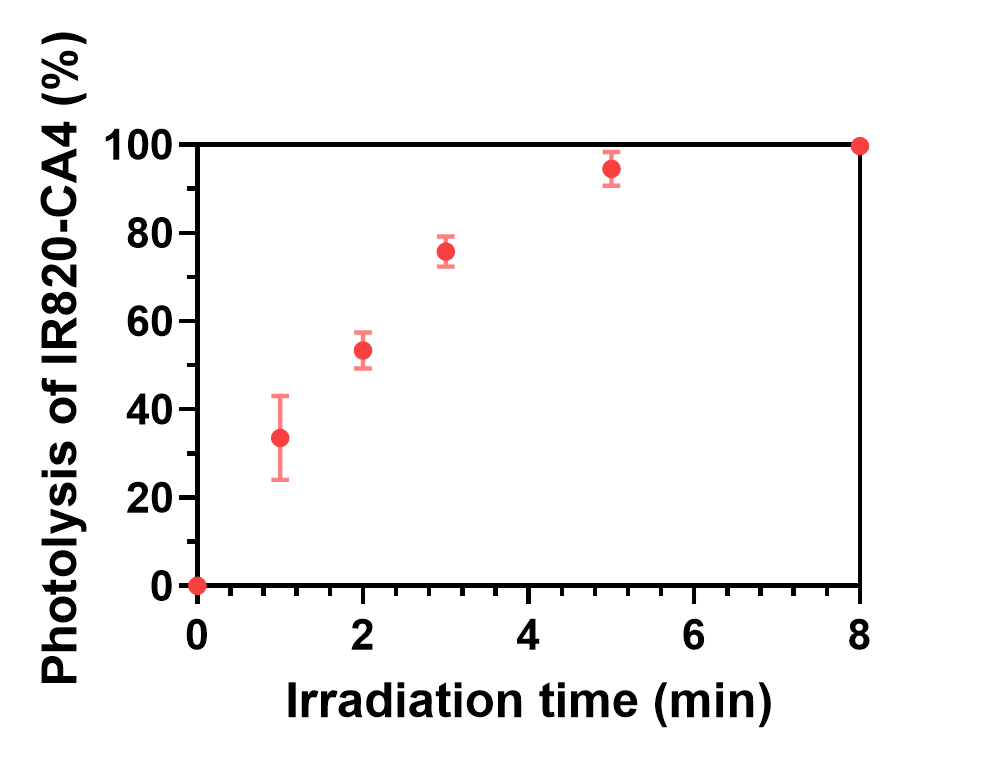
Figure S4. Quantitative analysis of photolysis percentages of IR820-CA4 upon 690 nm light irradiation (80 mW/cm^2^) for various periods. Data were presented as mean ± standard deviation. n =3.


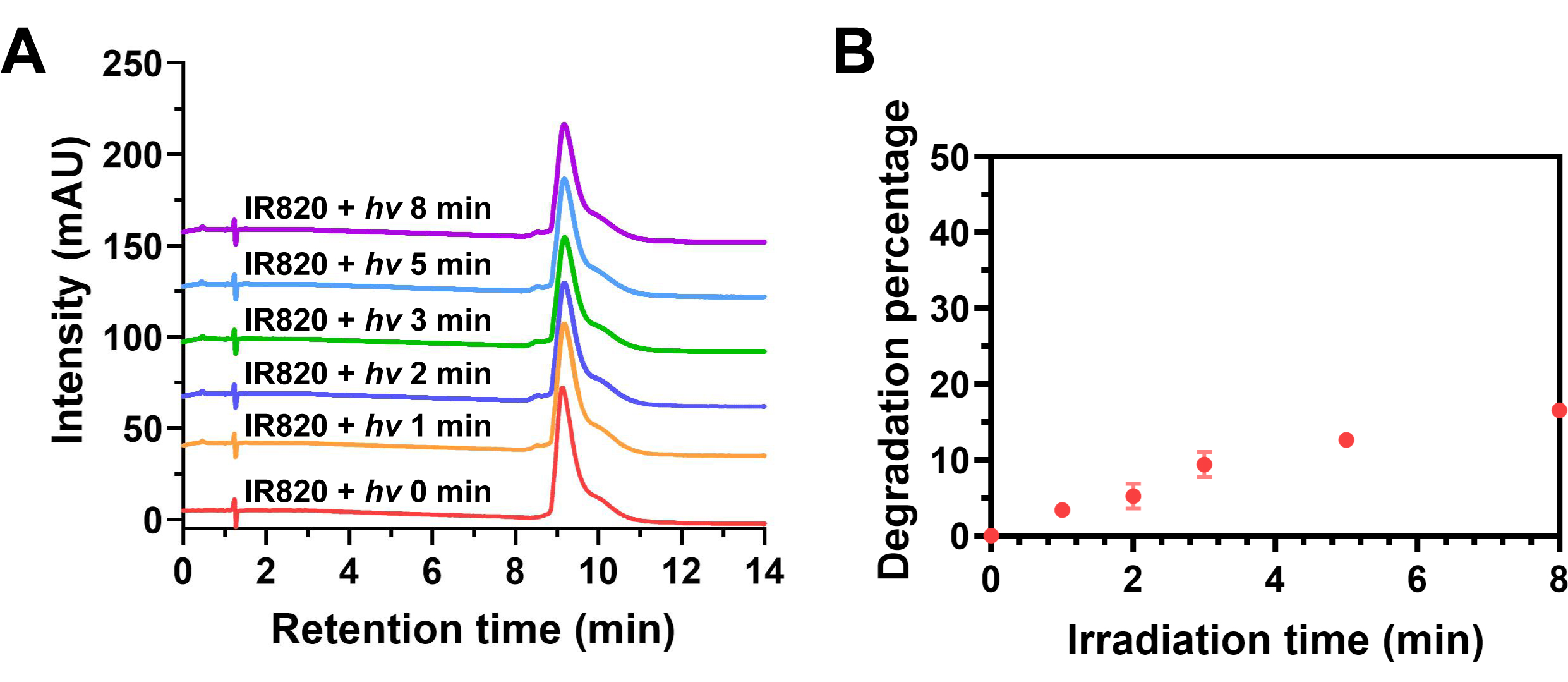


Figure S5. (A) Representative HPLC chromatograms and (B) quantitative analysis of degradation percentages of IR820 after receiving different periods of 690 nm light irradiation (80 mW/cm^2^). The results suggested a better photostability of IR820 compared to IR820-CA4 prodrug under 690 nm light irradiation.


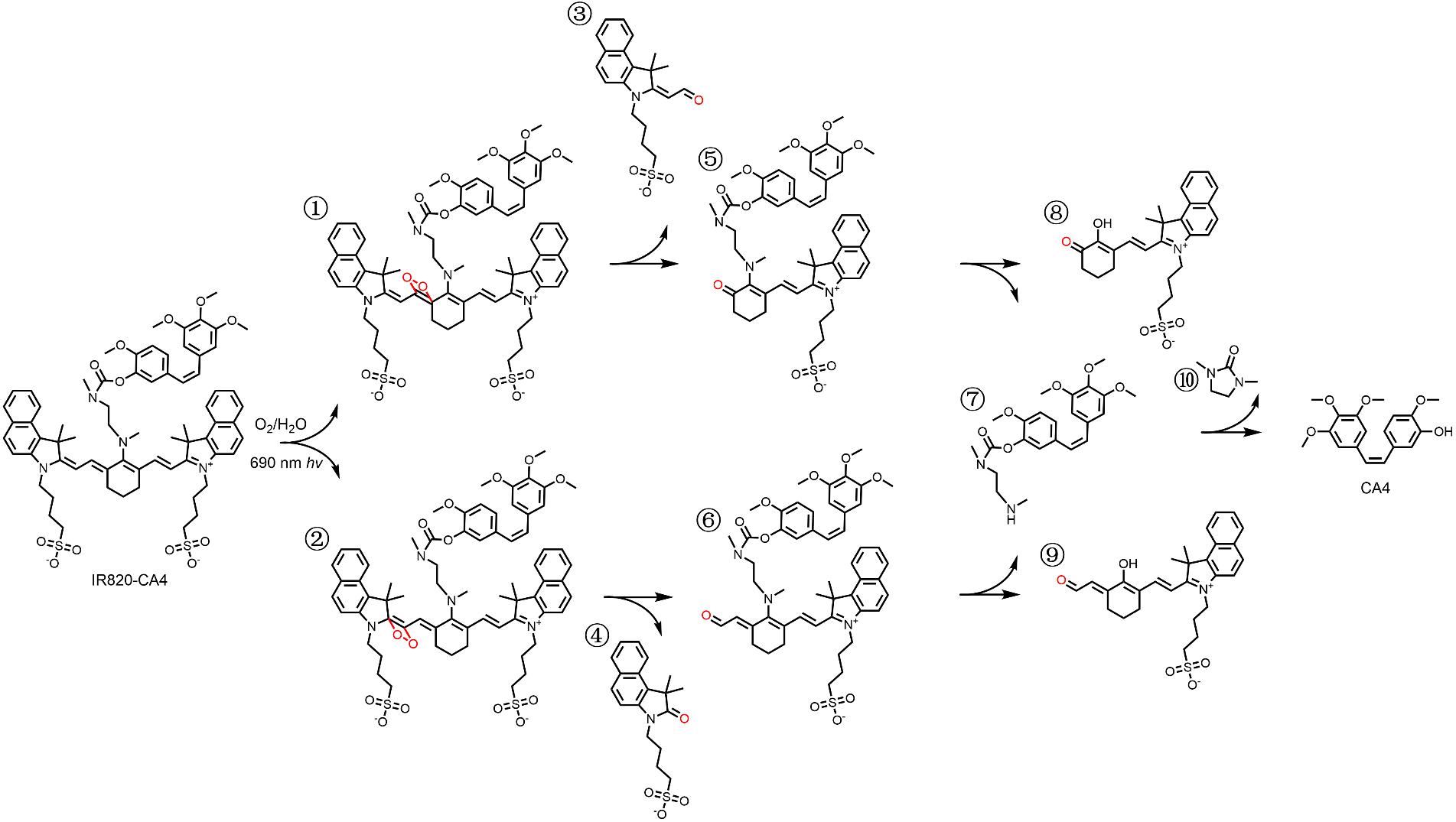


Figure S6. Schematic illustration of the mechanism of near-infrared (NIR) light-triggered degradation of IR820-CA4 prodrug to release CA4.


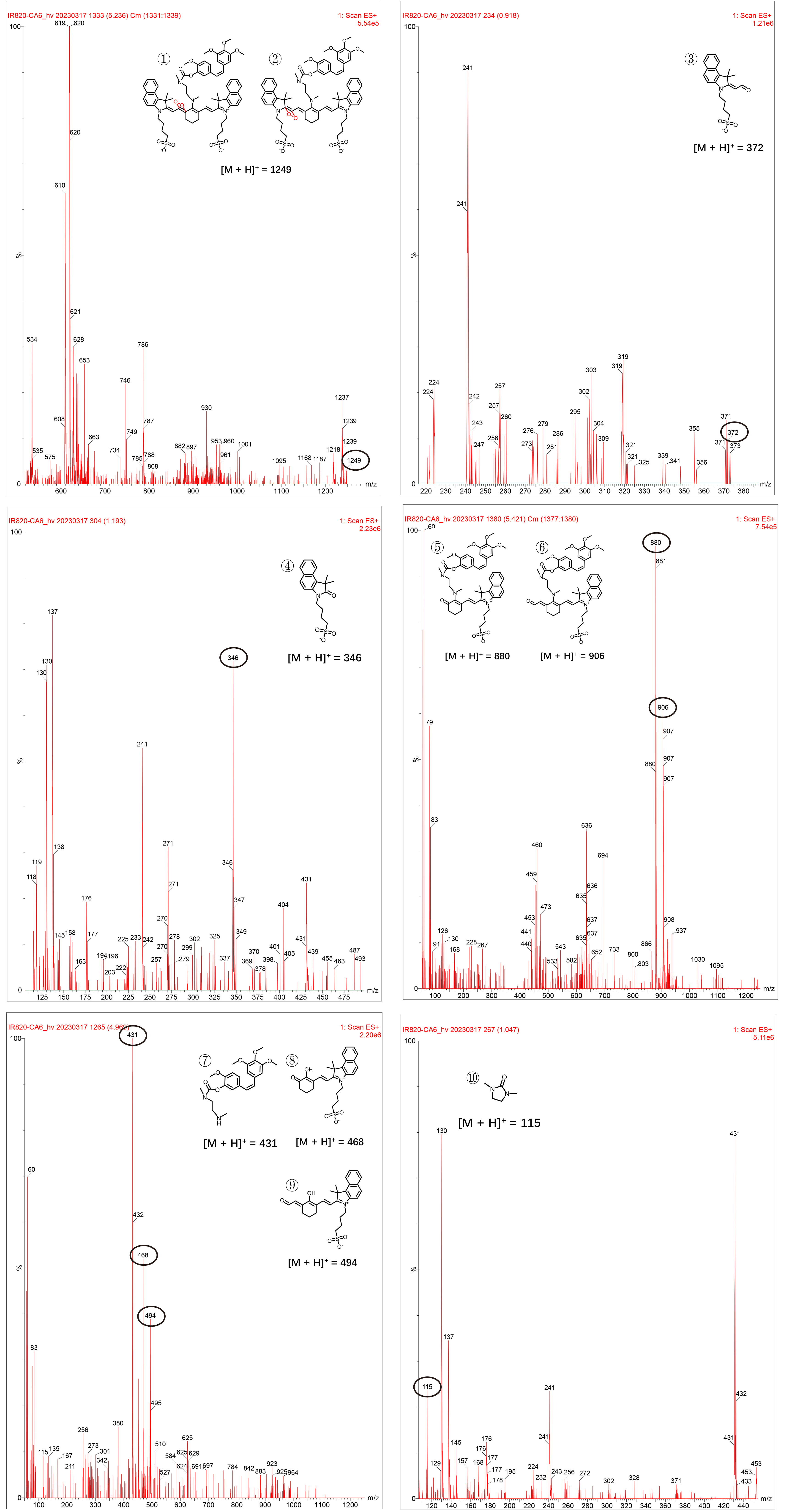


Figure S7. ESI-MS spectra of photolyzed products of IR820-CA4. Peaks of [M+H]^+^ are denoted by black circles.


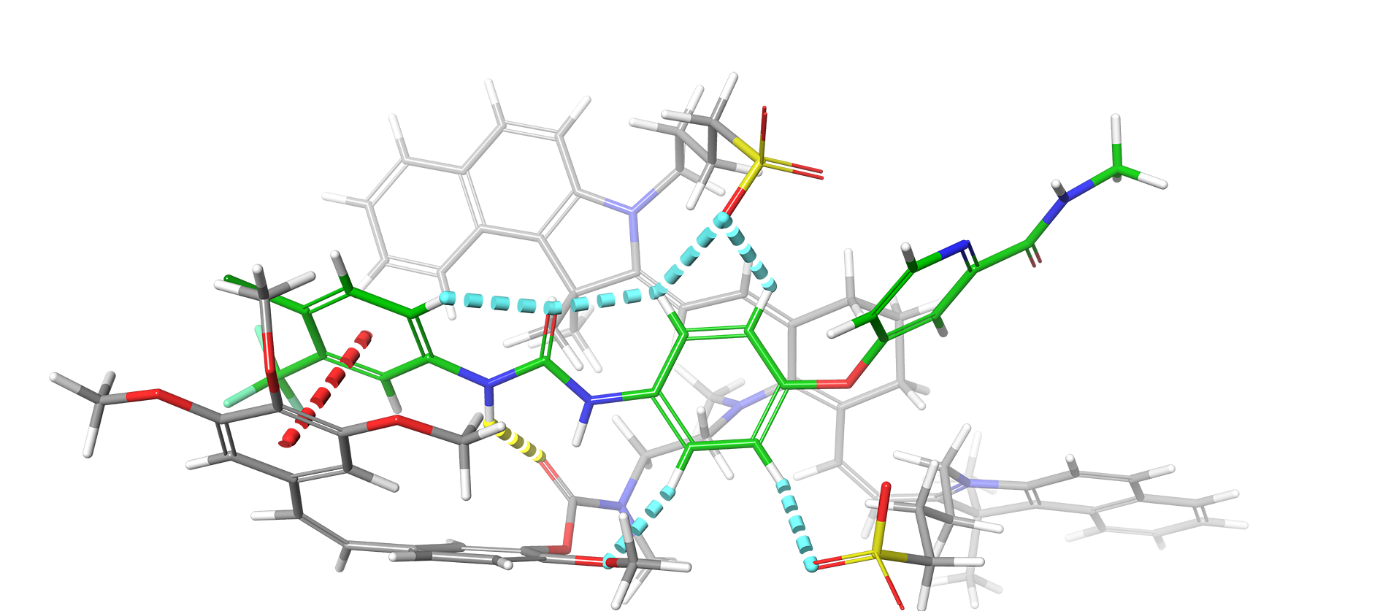


Figure S8. Schematic illustration of intermolecular interactions between SOR molecule (green backbone) and IR820-CA4 molecule (gray backbone). Red dash lines denote π-π stacking, yellow dash lines denote hydrogen bonds, and light blue dash lines denote aromatic hydrogen bonds. Molecular docking analysis was conducted with the ligand docking module of Schrodinger Maestro software (Release 2018-1).





Figure S9. Quantitative analysis of (A) intensity-based DLS size data, and (B) loading capacities and encapsulation efficiencies of IR820-CA4 in the formulations prepared with SOR and varying feeding ratio of IR820-CA4. The prodrug nanoparticles prepared with 5% of feeding ratio of IR820-CA4 to SOR showed desirable size distribution and relatively high IR820-CA4 loading capacity. Data were presented as mean ± standard deviation. n =3.

Table S1. Physiochemical properties of SOR/IR820-CA4 NPs and SOR/IR820 NPs. Data were presented as mean ± standard deviation. n =3.

|  | **Particle Size (nm)** | **PDI** | **Zeta Potential (mV)** | **IR820-CA4/IR820**  **Encapsulation Efficiency (%)** | **IR820-CA4/IR820-**  **Loading (%)** | **SOR**  **Encapsulation Efficiency (%)** | **SOR-**  **Loading (%)** |
| --- | --- | --- | --- | --- | --- | --- | --- |
| **SOR/IR820-CA4 NPs** | 124.2 ± 6.92 | 0.116 ± 0.019 | -29.4 ± 1.8 | 14.81 ± 0.595 | 2.86 ± 0.131 | 26.50 ± 0.194 | 97.14 ± 0.131 |
| **SOR/IR820 NPs** | 108.3 ± 4.76 | 0.136 ± 0.016 | -28.5 ± 1.26 | 19.73 ± 1.427 | 3.47 ± 0.303 | 27.47 ± 0.554 | 96.52 ± 0.303 |


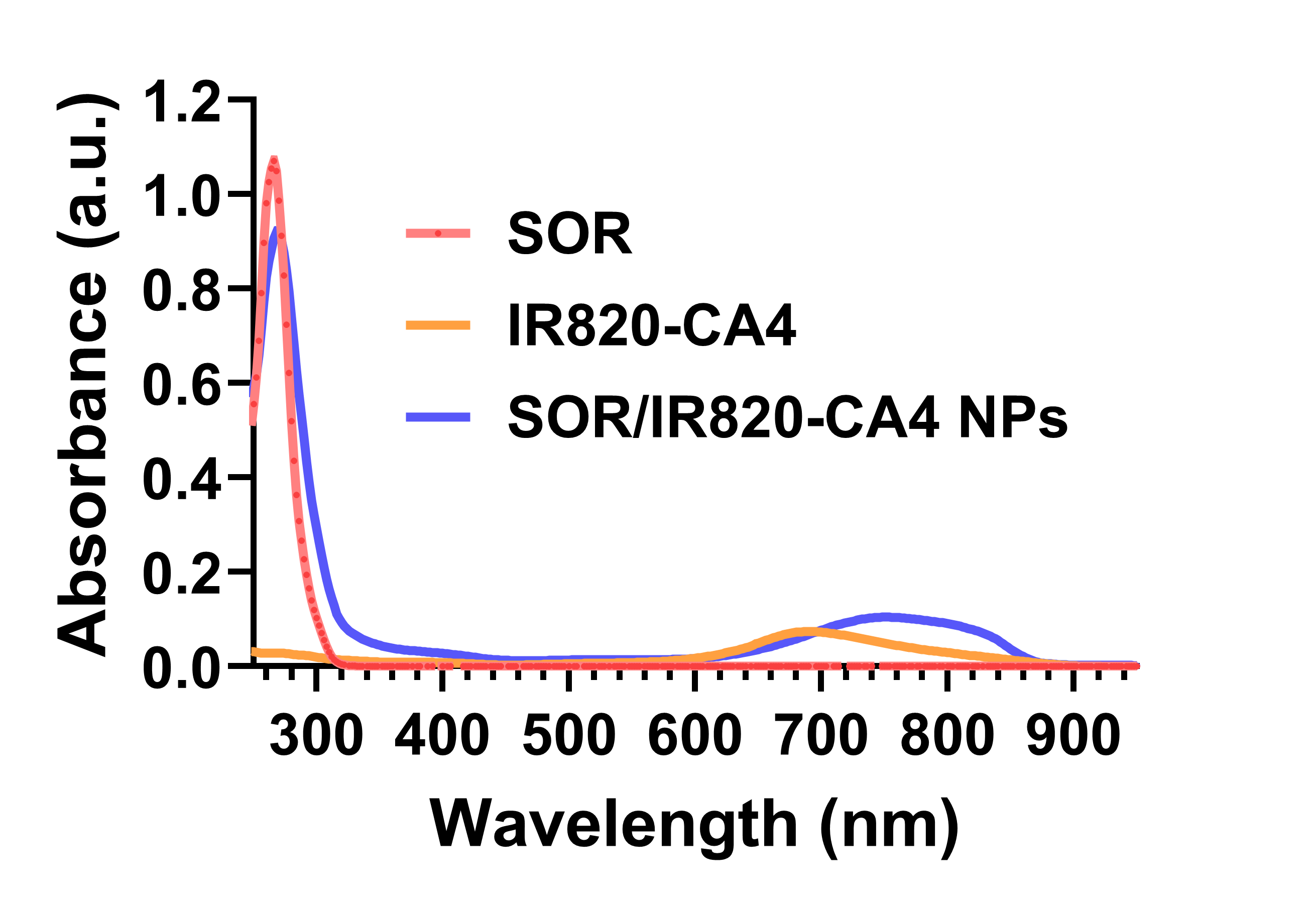
Figure S10. Representative absorption spectra of SOR, IR820-CA4 and the prodrug nanoparticles.


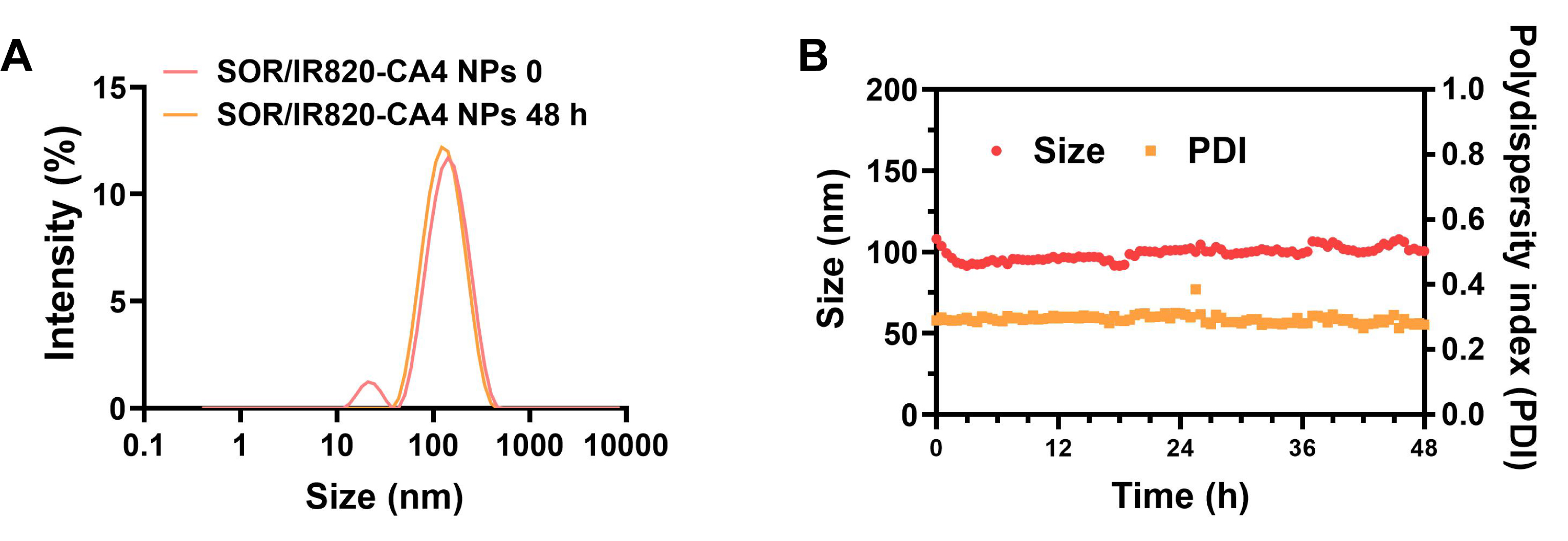
Figure S11. (A) Representative size distribution and (B) DLS size and polydispersity index (PDI) data of SOR/IR820-CA4 NPs after incubation in DMEM medium supplemented with 10% fetal bovine serum (FBS) at 37 ^o^C for 48 h.


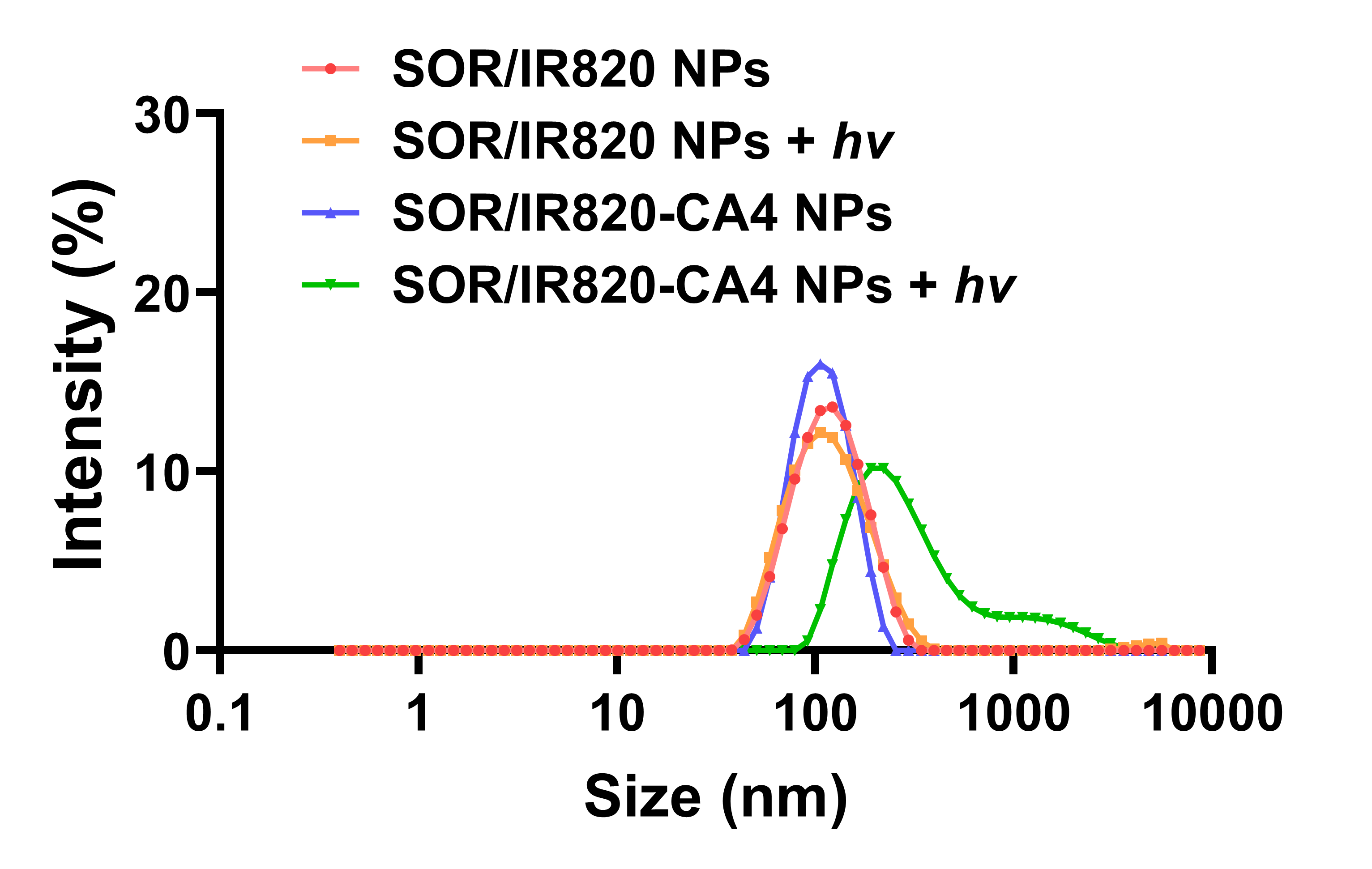
Figure S12. Representative images of size distribution profiles of SOR/IR820 NPs and SOR/IR820-CA4 NPs before and after 690 nm laser irradiation (80 mW/cm^2^, 10 min).


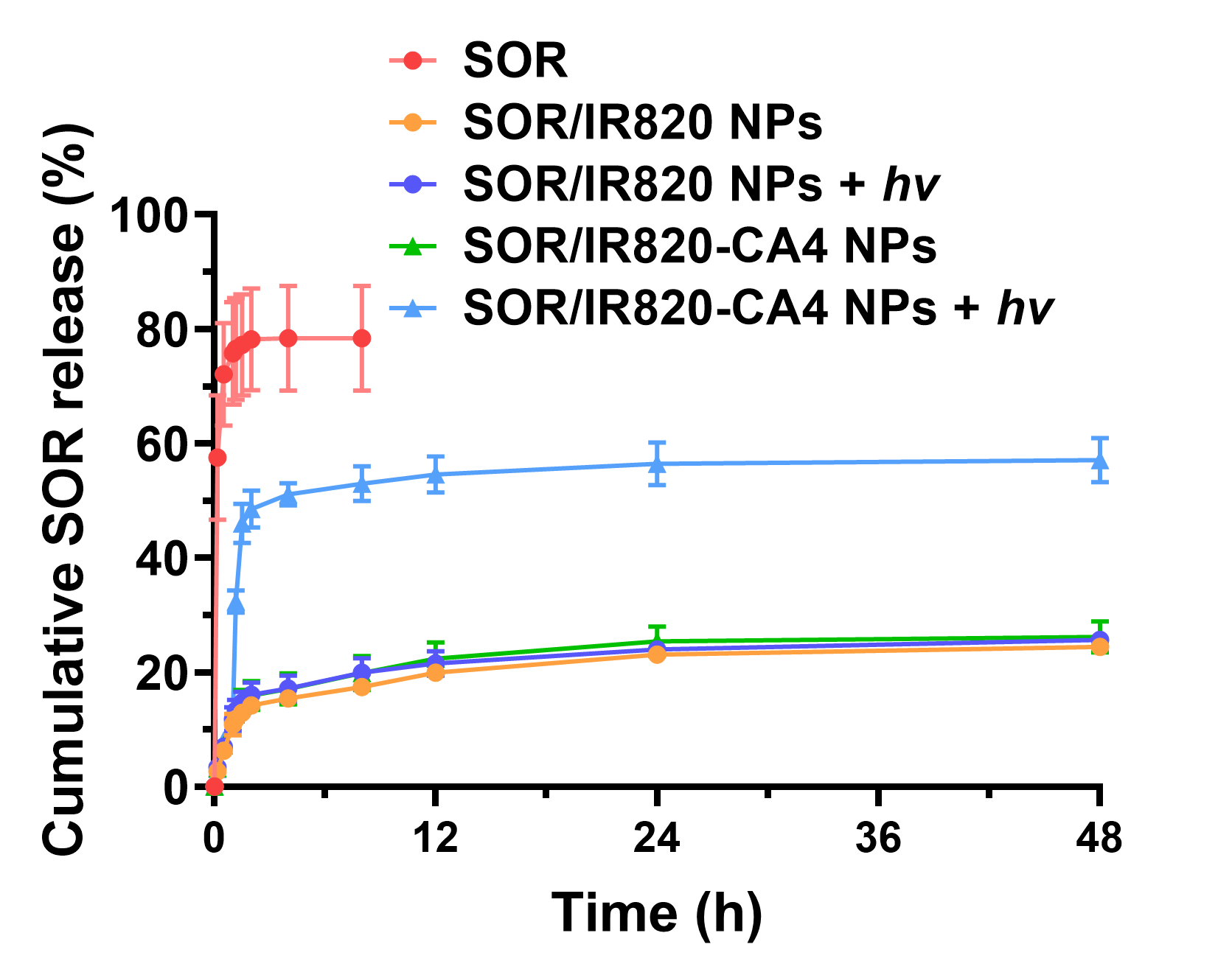
Figure S13. Cumulative SOR release profiles of SOR/IR820-CA4 NPs or SOR/IR820 NPs. The nanoparticle solutions were exposed to NIR light irradiation (690 nm, 80 mW/cm^2^, 5 min) at 1h or not. Data were presented as mean ± standard deviation. n =3.


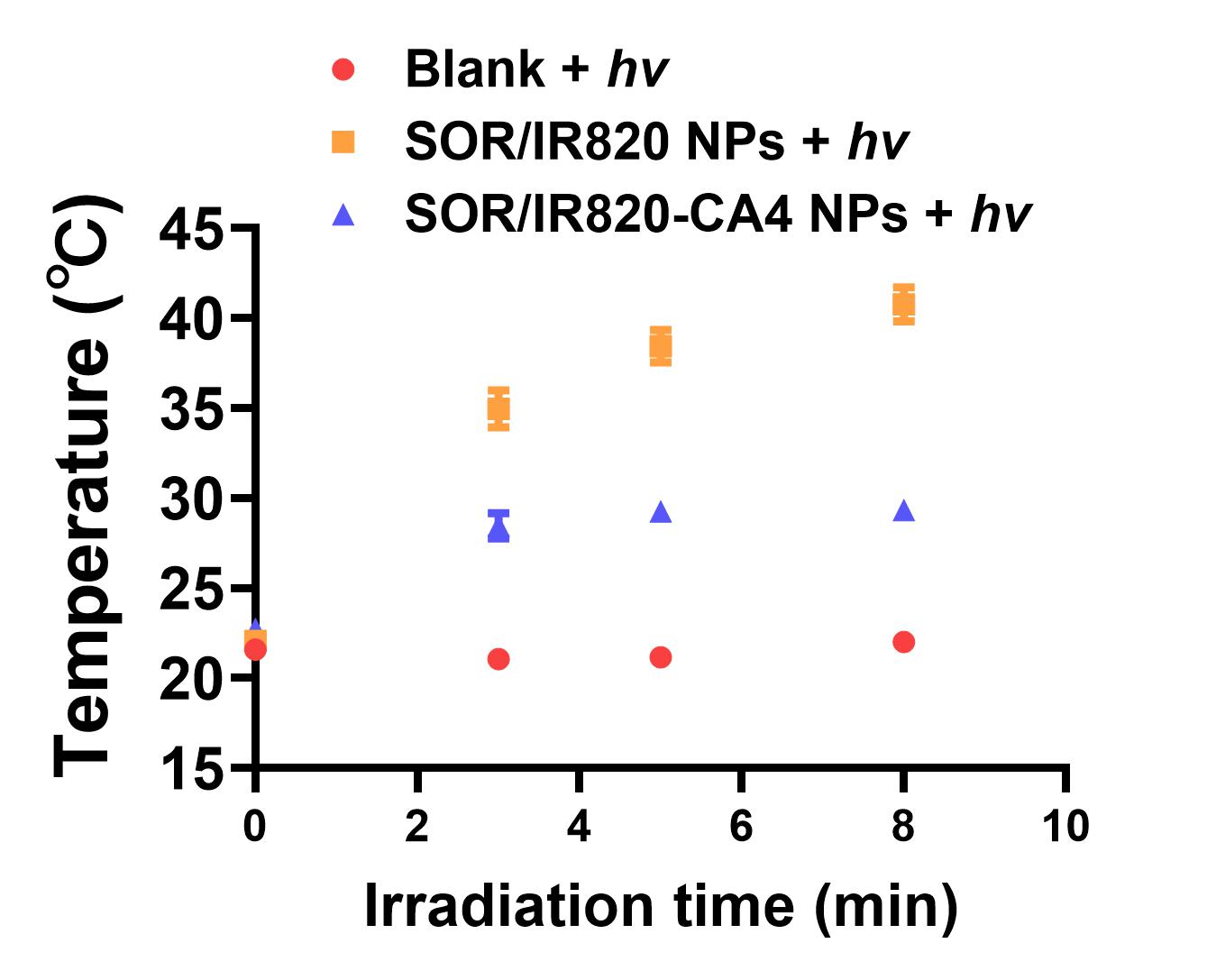
Figure S14. Quantitative analysis of temperature variations of blank solvent, SOR/IR820 NPs and SOR/IR820-CA4 NPs irradiated by a 690 nm laser (80 mW/cm^2^). Data were presented as mean ± standard deviation. n =3.



Figure S15. (A) Representative flow cytometric histograms displaying HUVEC uptake of IR820-CA4 or SOR/IR820-CA4 NPs at 4 h post-incubation. (B) Quantitative analysis of the fluorescence intensity of IR820-CA4 in HUVECs after 4 h incubation with different formulations. Data were presented as mean ± standard deviation. n =3. *** *p* < 0.001.


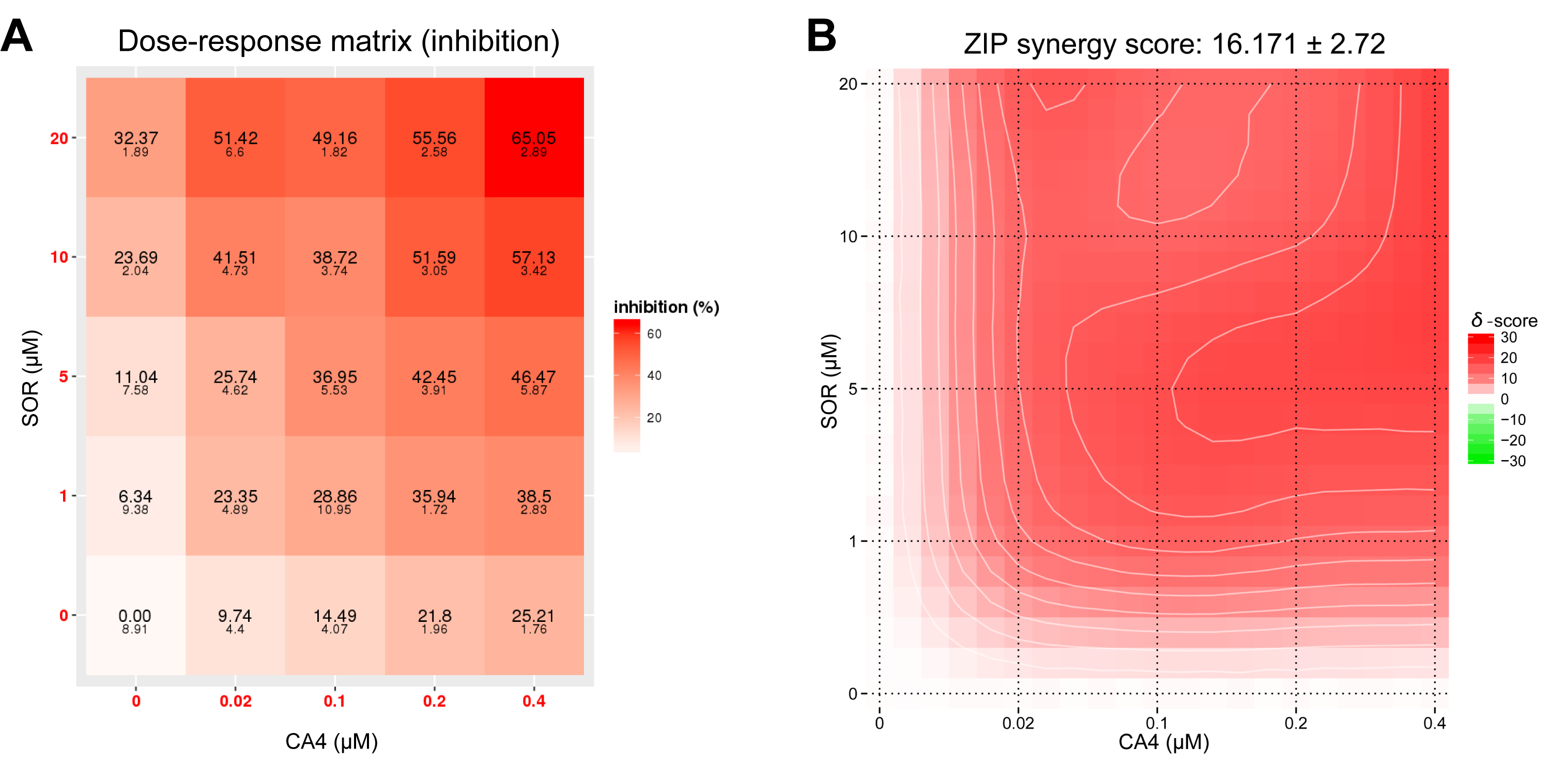
Figure S16. *In vitro* synergistic effect evaluation of SOR and CA4. (A) Quantitative analysis of cell death percentages of HUVECs subject to varying concentrations of SOR and CA4. Cells were treated with 20 ng/mL VEGF_165_ and the indicated drugs. MTT assay was performed at 24 h post-treatment. Data were presented as the mean ± SD. n = 6. (B) Synergy score map corresponding to (A), calculated by SynergyFinder Plus with Zero interaction potency (ZIP) mode. ZIP synergy score > 10 indicates the potential synergistic effect.


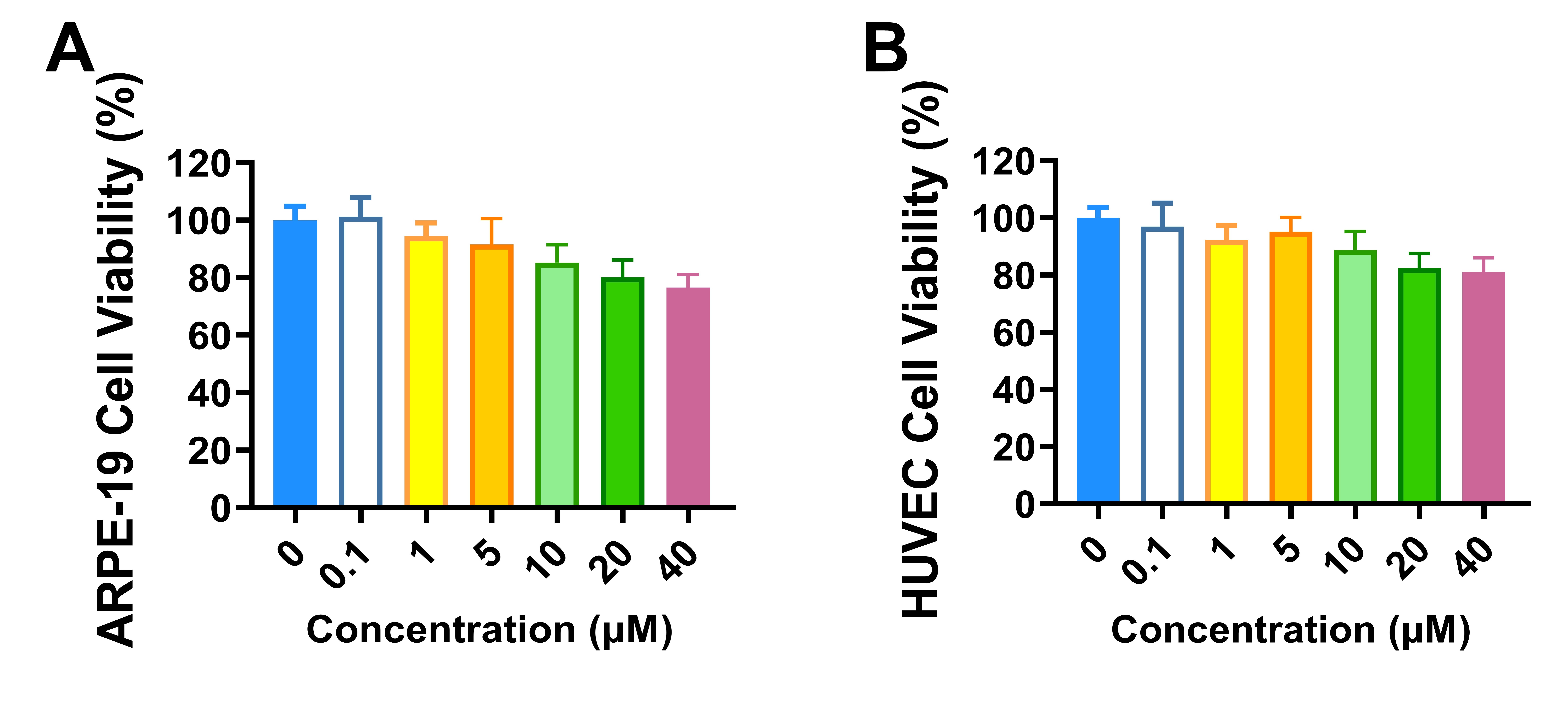
Figure S17. Quantitative analysis of (A) ARPE-19 cell viability and (B) HUVEC cell viability after being treated with varying concentrations of SOR/IR820-CA4 NPs for 48 h. Data were presented as the mean ± SD. n = 6.


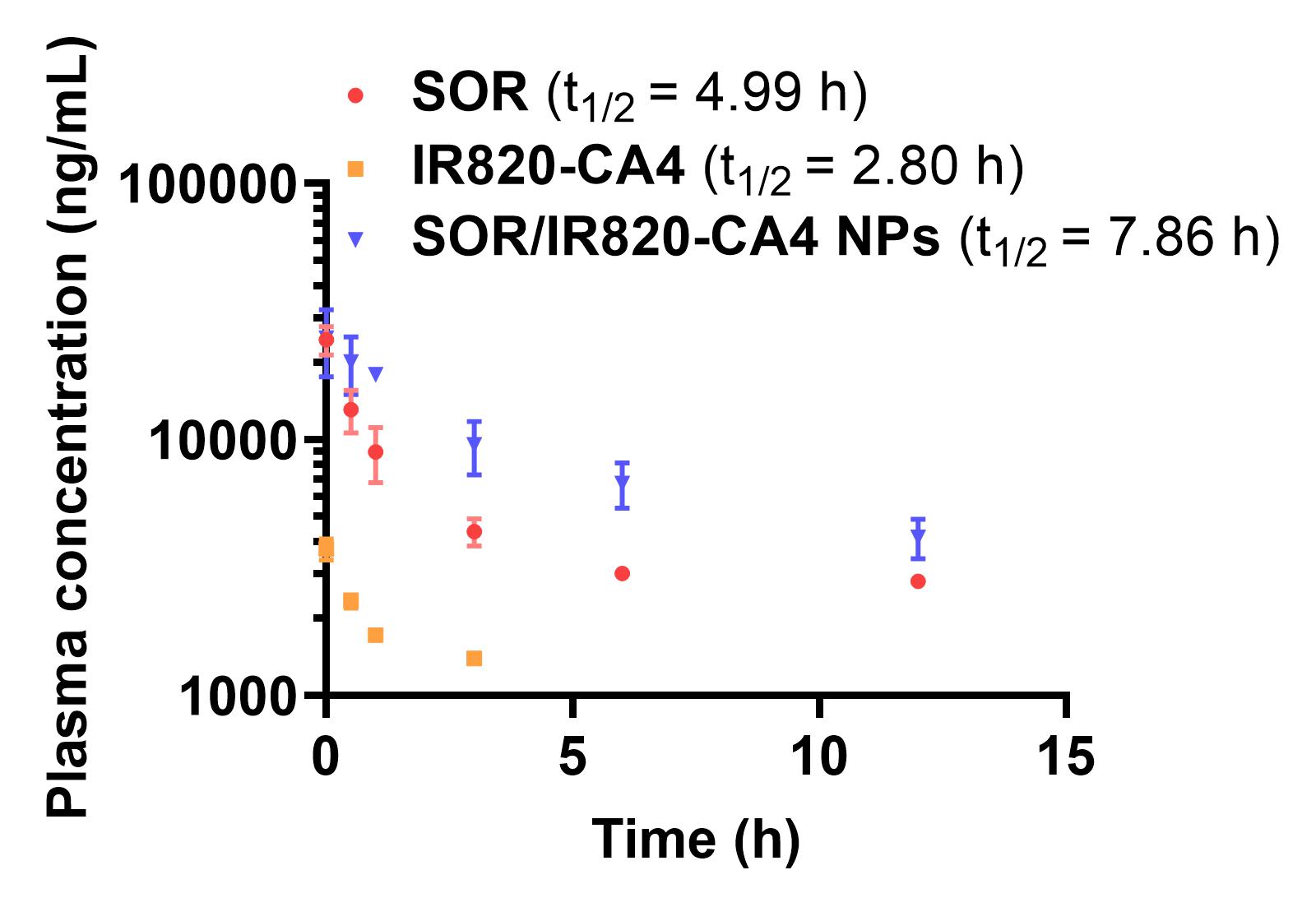


Figure S18. Plasma concentration versus time profile in C57BL/6 mice following a single intravenous injection of different formulations at the equivalent dose of 10 mg/kg SOR. The administration dose of IR820-CA4 was the same as that of SOR/IR820-CA4 NPs determined by HPLC. Data were presented as the mean ± SD. n = 3.

Table S2. Pharmacokinetic parameters of different formulations in C57BL/6 mice after a single intravenous injection of different formulations.

| **Parameter** | **SOR** | **IR820-CA4** | **SOR/IR820-CA4 NPs** |
| --- | --- | --- | --- |
| t_1/2_ (h) | 4.99 ± 0.94 | 2.80 ± 0.33 | 7.86 ± 1.02 |
| C_max_ (ng/mL) | 24528.25 ± 2527.20 | 3737.80 ± 287.62 | 25550.41 ± 5568.00 |
| AUC_0-t_ (ng⋅h/mL) | 56744.48 ± 3288.95 | 5670.53 ± 160.06 | 106515.90 ± 14514.16 |
| CL_obs_ (mL/h/kg) | 130.36 ± 4.49 | 26.22 ± 1.91 | 65.71 ± 7.07 |
| MRT_0-inf_obs_ (h) | 8.05 ± 1.06 | 2.80 ± 0.32 | 10.16 ± 1.31 |

Note: t_1/2_, plasma terminal elimination half-life; C_max_, maximum observed plasma concentration; AUC_0-t_, area under the drug concentration-time curve from time 0 to the last observed time t; Cl_obs_, apparent systemic clearance of the drug; MRT_0-inf_obs_, apparent mean residence time of drug. Plasma concentration data were analyzed using PKSolver with noncompartmental data analysis mode. Data were presented as mean ± SD. n =3. SOR, sorafenib.

Table S3. Quantitative analysis of SOR concentration in C57BL/6 mice at 1 h post-intravenous injection of different formulations.

|  | **Choroid-RPE** | **Retina** | **Other ocular tissue** |
| --- | --- | --- | --- |
| **SOR** | 335.17 ± 191.05 | 398.73 ± 122.09 | 84.49 ± 46.24 |
| **SOR/IR820-CA4 NPs** | 719.52 ± 56.05 | 678.87 ± 141.83 | 162.69 ± 28.17 |

Data were presented as mean ± SD (unit: ng/g). n =3. RPE, retinal pigment epithelium; SOR, sorafenib.


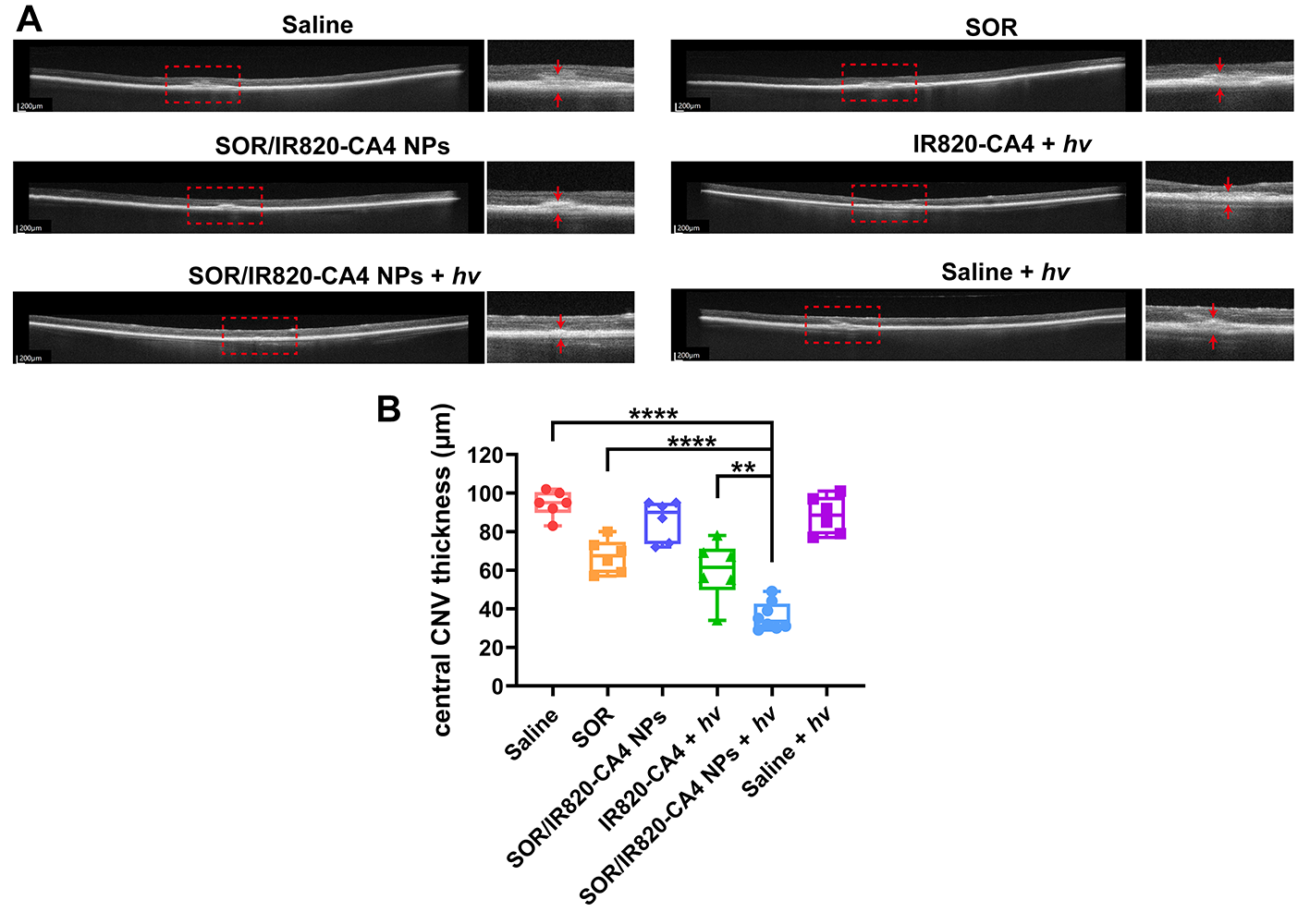
Figure S19. (A) Representative cross-sectional optical coherence tomography (OCT) images of CNV lesions of mice after different formulation treatments. Magnified images of CNV lesions are shown on the right panel of the original images (denoted by red dashed box). (B) Quantitative analysis of central CNV thickness of mice after different formulation treatments. Data were presented as the mean ± SD. n = 6. ** *p* < 0.01.***; *p* < 0.0001.
